# Pharmacological boost of DNA damage response and repair by enhanced biogenesis of DNA damage response RNAs

**DOI:** 10.1038/s41598-019-42892-6

**Published:** 2019-04-23

**Authors:** Ubaldo Gioia, Sofia Francia, Matteo Cabrini, Silvia Brambillasca, Flavia Michelini, Corey W. Jones-Weinert, Fabrizio d’Adda di Fagagna

**Affiliations:** 1 IFOM – the FIRC Institute of Molecular Oncology, Via Adamello 16, 20139 Milan, Italy; 2 Istituto di Genetica Molecolare, Luigi Luca Cavalli-Sforza - Consiglio Nazionale delle Ricerche, 27100 Pavia, Italy; 3 Present Address: Human Oncology and Pathogenesis Program (HOPP) - Memorial Sloan Kettering Cancer Center, New York, NY 10065 USA

**Keywords:** Double-strand DNA breaks, Small RNAs

## Abstract

A novel class of small non-coding RNAs called DNA damage response RNAs (DDRNAs) generated at DNA double-strand breaks (DSBs) in a DROSHA- and DICER-dependent manner has been shown to regulate the DNA damage response (DDR). Similar molecules were also reported to guide DNA repair. Here, we show that DDR activation and DNA repair can be pharmacologically boosted by acting on such non-coding RNAs. Cells treated with enoxacin, a compound previously demonstrated to augment DICER activity, show stronger DDR signalling and faster DNA repair upon exposure to ionizing radiations compared to vehicle-only treated cells. Enoxacin stimulates DDRNA production at chromosomal DSBs and at dysfunctional telomeres, which in turn promotes 53BP1 accumulation at damaged sites, therefore in a miRNA-independent manner. Increased 53BP1 occupancy at DNA lesions induced by enoxacin ultimately suppresses homologous recombination, channelling DNA repair towards faster and more accurate non-homologous end-joining, including in post-mitotic primary neurons. Notably, augmented DNA repair stimulated by enoxacin increases the survival also of cancer cells treated with chemotherapeutic agents.

## Introduction

DNA double-strand breaks (DSBs) are harmful genomic lesions that need to be promptly recognized to ensure a fast and efficient repair. The DNA damage response (DDR) is a tightly regulated signalling cascade that senses genotoxic insults, amplifies and propagates the signals, and imposes a cell cycle arrest to allow DNA repair to take place and preserve genome integrity^1^. In this cascade of events, the MRE11-RAD50-NBS1 (MRN) complex is one of the first factors to directly bind to DSBs, locally triggering the auto-phosphorylation and activation of the ATM kinase, in a process called primary recruitment^2,3^. The activated form of ATM (pATM^S1981^) then phosphorylates the C-terminal portion of histone H2AX (γH2AX), allowing the recruitment of the DDR mediator factors MDC1 and 53BP1 (TP53BP1) as well as more MRN-ATM complexes. This process is also known as secondary recruitment and functions as a positive feedback loop which amplifies the DDR signal and spreads the accumulation of DDR factors for hundreds of kilobases starting from the DSB^3,4^. The signal is ultimately transduced from the apical kinase ATM to downstream kinases such as CHK2, thus enforcing checkpoint activation^5^.

In the last few years, a novel class of small non-coding RNAs (sncRNAs), that are generated at DNA damage sites in a DROSHA- and DICER-dependent manner, was discovered and shown to be critical for the full activation of DDR pathways in higher eukaryotes. These short molecules, named DNA damage response RNAs (DDRNAs), are required to mount the DDR amplification cascade by favouring the secondary recruitment of DDR factors at chromosomal DSBs^3,6^ as well as at dysfunctional telomeres in cultured cells and *in vivo*^7^. More recently, our group demonstrated that nucleation of DDR factors to DSBs occurs through their interaction with DDRNAs and their precursors^8^. The DSB-induced RNAs (diRNAs), that are similar to DDRNAs, have been proposed to be involved in DNA repair both by homologous recombination (HR) and non-homologous end joining (NHEJ)^9–11^.

The biogenesis and function of this novel class of sncRNAs relies on specific components of the RNA interference (RNAi) machinery, directly linking their canonical roles in gene silencing to DDR modulation and genome stability^12,13^. In particular, the phosphorylated form of DICER has been reported to accumulate at DSBs where it catalyses damage-induced RNA processing^14^. In this regard, loss of RNAi components has been associated to DNA damage accumulation and increased genome instability in mammals^14–16^.

Enoxacin is a small molecule belonging to the fluoroquinolone family of antibiotics that was recently found to promote DICER activity^17,18^. To exert its function, DICER interacts with both the Protein Activator of the Interferon-induced Protein Kinase (PACT) and TRBP (HIV TAR RNA-binding protein, encoded by TARBP2 gene), two double-stranded RNA-binding proteins that are required for proper miRNA maturation^19–22^. Specifically, enoxacin facilitates the binding of TRBP to its RNA substrates, thus enhancing DICER-directed RNA processing^17,18^. Importantly, enoxacin has been proposed to be a potential anti-cancer drug that acts by increasing the levels of a subset of mature tumour suppressing miRNAs^18,23^ and, more recently, to ameliorate neuromuscular function *in vivo* in an Amyotrophic Lateral Sclerosis (ALS) mouse model by improving miRNA processing^24^.

Here, we unveil a previously unknown miRNA-independent function of enoxacin and demonstrate that DDR and DNA repair can be enhanced pharmacologically by enoxacin through its ability to stimulate DDRNA biogenesis. We show that the elevated DDRNA levels triggered by enoxacin promote 53BP1 recruitment to sites of damage, thus accelerating DNA repair by NHEJ and ultimately increasing cell survival following exogenous DNA damage. To date, this represents the first approach to potentiate DDR and DNA repair in cultured cells by acting on an RNA processing mechanism.

## Results

### Enoxacin boosts DDR via TRBP activity

**Figure 1:**
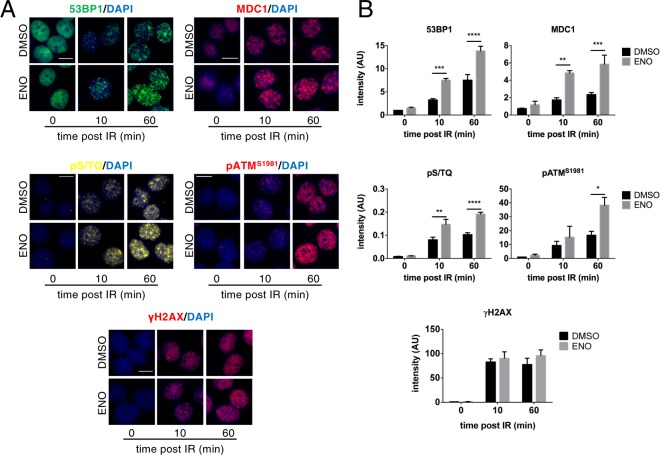
Enoxacin boosts DDR signalling. (**A**) HeLa cells, treated with enoxacin (ENO) or DMSO for 48 hours, were fixed at the indicated time points post IR and immuno-stained for 53BP1 (green), pS/TQ (yellow), MDC1, pATM^S1981^ or γH2AX (red); nuclei were counter-stained with DAPI (blue). As a control, not irradiated cells are shown (0 panels). Scale bars = 10 µm. (**B**) Quantification of DDR activation represented in (**A**); the intensity of DDR foci per nucleus is shown for each time point; values are the means ± s.e.m. of at least three independent experiments; at least 300 cells per sample were scored. 0 min post IR refers to not irradiated cells.

**Figure 2:**
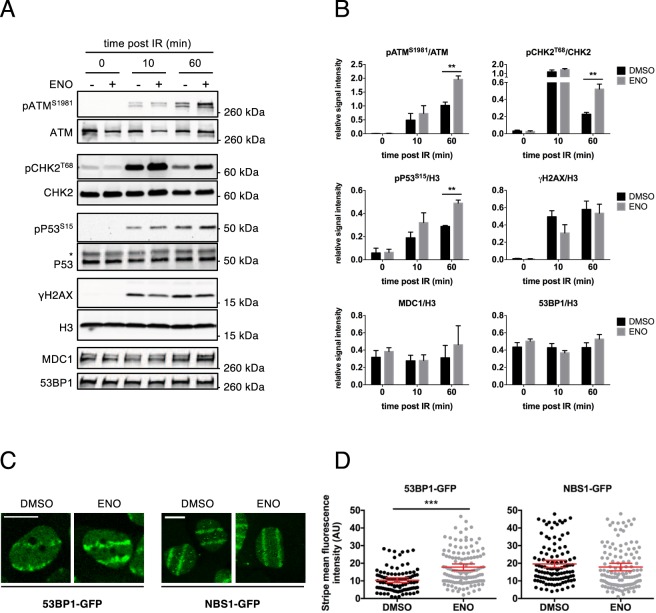
Enoxacin enforces ATM-CHK2-P53 signalling axis and enhances secondary recruitment of DDR factors. (**A**) HeLa cell whole lysates were analysed for the indicated proteins by western blot; the asterisk marks unspecific signals (cell conditions as in Fig. 1). (**B**) Densitometric analysis of protein levels shown in (**A**); values are the averages ± s.e.m. of at least three independent experiments (Student’s t-test). 0 min post IR refers to not irradiated cells. (**C**) Representative images of laser micro-irradiated cells expressing 53BP1-GFP or NBS1-GFP and treated with enoxacin or DMSO for 48 hours. Scale bars = 10 µm. (**D**) Quantification of DDR fluorescence intensity at laser stripes shown in (**C**). The plot shows the distribution of 53BP1-GFP or NBS1-GFP laser-stripe fluorescence intensity measured at 15 min. Red bars indicate the average values ±95% confidence interval (CI) from three independent experiments; at least 100 cells were measured for each condition (Student’s t-test).

Since it has been previously shown that DICER endoribonuclease activity is crucial for DDR activation^3,6,25,26^, we tested whether the enhancement of DICER processing by a pharmacological treatment could promote DDR activation. Thus, we treated HeLa cells with 50 μM enoxacin (or DMSO as vehicle-only control) for 48 hours before exposure to ionizing radiation (IR). We then analysed the activation of DDR at different time points after IR by quantitative immunofluorescence (IF) for γH2AX, pATM^S1981^, 53BP1, MDC1 and pS/TQ (the substrate of active ATM). Cells treated with enoxacin prior to IR mounted stronger DDR activation than control cells treated with DMSO, as measured by the intensity of DDR foci per nucleus (Fig. 1). The observed unaltered γH2AX levels within 1 hour post IR (Fig. 1) are in line with conclusions published by us and others^3,6,11^ and confirm equal initial amounts of DNA damage induction among samples. Importantly, enoxacin did not increase the expression of the proteins studied, as detected by immunoblotting of whole cell lysates: this indicates that their activation, rather than their expression levels, is affected by enoxacin treatment (Fig. 2A,B). To evaluate the dose-dependency of enoxacin-mediated DDR boost, we treated HeLa cells with increasing concentrations of the drug (50, 100 and 200 μM). Since administration of high doses of enoxacin (>50 μM) for more than 1 day is detrimental for cancer cell viability^18,23^, we incubated cells for 24 hours before IR and probed for DDR factors including 53BP1, pS/TQ, pATM^S1981^ and γH2AX by quantitative immunofluorescence. We observed a good dose response of 53BP1 and pS/TQ activation up to 100 μM of enoxacin dosage while γH2AX levels were substantially unchanged (Fig. S1). Notably, pATM activation peaked at 50 μM to proportionally decrease at higher drug concentrations (Fig. S1). This is consistent with a dose-dependent effect of enoxacin on miRNAs targeting ATM mRNA^6,27–29^, consequently reducing its protein levels as confirmed by immunoblotting on total cell lysates (Fig. S2A,B). This reduction may account for the decrease of 53BP1 recruitment and pS/TQ activation observed at the highest enoxacin dosage (200 μM) (Fig. S1). As such, we used the 50 μM concentration for our subsequent DDR analysis.

Next, we tested whether the increase in DDR foci intensity was only the result of a more efficient DDR protein accumulation at sites of DNA damage or also the consequence of their augmented activation. Thus, we monitored the levels of the activated form of ATM (pATM^S1981^) in whole cell lysates by immunoblotting. We found that enoxacin augmented pATM^S1981^ levels indicating that ATM activation has been enhanced by enoxacin (Figs 2A,B and S2A,B). Moreover, immunoblot analyses independently confirmed results obtained by IF for γH2AX and pATM^S1981^ (Figs 1 and 2A,B).

Starting from the DDR focus, signalling spreads by engaging downstream protein kinases that are only transiently localized to sites of DNA damage and then diffuse freely throughout the nucleoplasm, ultimately leading to cell cycle checkpoint activation^5,30^. We thus tested if the activation of the main ATM substrates, such as the effector kinase CHK2 and P53, were also affected by enoxacin treatment. We observed that enoxacin boosts their activation too, as assessed by increased levels of their phosphorylated forms, pCHK2^T68^ and pP53^S15^, thus demonstrating the full activation of the ATM-CHK2-P53 signalling axis (Fig. 2A,B).

We recently demonstrated that DDRNAs are dispensable for primary recognition of DSBs, being instead involved in the subsequent DDR amplification^3^. To further investigate the mechanism of action of enoxacin in DDR activation, we tested its impact on primary and secondary recruitment of DDR factors by using two previously validated U2OS cell lines stably expressing GFP-fused versions of NBS1 or 53BP1, as readout of primary and secondary recruitment respectively^31,32^. Cells were pre-sensitized with BrdU and subjected to UVA-laser micro-irradiation, as previously described^3^. 48 hours post-enoxacin administration, we observed that 53BP1-GFP recruitment kinetics to stripes of laser micro-irradiation was significantly enhanced as early as 8 min (*p* ≤ 0.022) post DNA damage induction, while NBS1-GFP localization did not show any increase (Figs 2C,D and S2C). Together, these results demonstrate that enoxacin impacts on DDR by stimulating secondary recruitment of DDR factors to sites of DNA damage.

**Figure 3:**
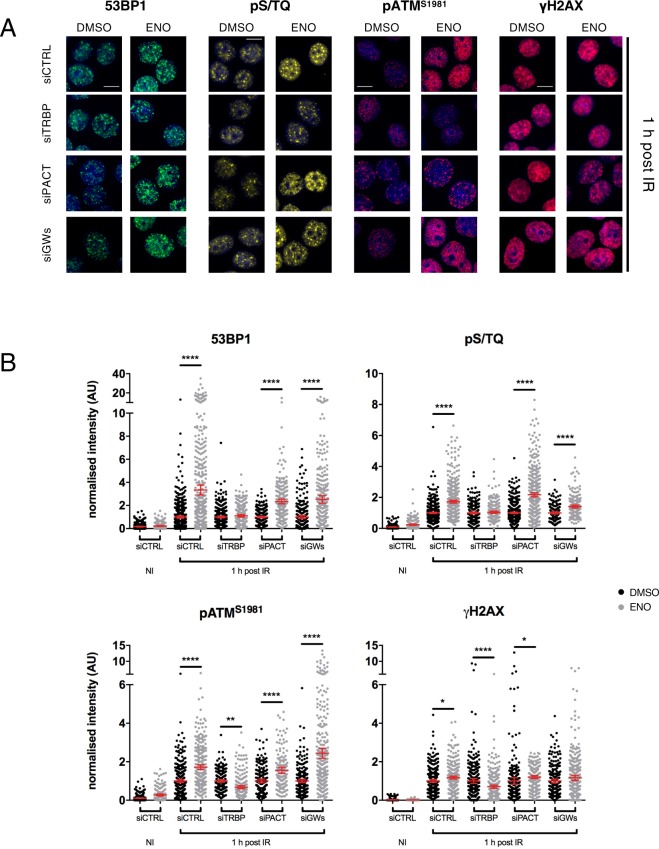
DDR activation mediated by enoxacin requires TRBP and is miRNA-independent. (**A**) HeLa cells were transfected with siRNAs against TRBP (siTRBP), PACT (siPACT), GW182 proteins (siGWs) or with a non-targeting siRNA (siCTRL) and simultaneously treated with enoxacin (ENO) or DMSO for 48 hours prior to irradiation. 1 hour post IR, cells were fixed and stained for 53BP1, pS/TQ, pATM^S1981^ or γH2AX. Scale bars = 10 µm. (**B**) Quantification of DDR activation shown in (**A**). Dot-plots show the intensity of DDR foci per nucleus from samples treated with enoxacin (grey dots) or DMSO (black dots). Values are relative to irradiated cells treated with DMSO (1 h post IR, black dots); red bars indicate the average values ±95% CI from three independent experiments; at least 200 cells were scored for each condition. NI = not irradiated samples.

Enoxacin has been proposed to stimulate DICER endoribonuclease activity by directly interacting with TRBP and promoting its binding to the RNA substrate^17,18^. We thus investigated the target specificity of enoxacin-mediated DDR enhancement by knocking down TRBP and PACT in HeLa cells treated or not with enoxacin for 48 hours before IR exposure. DDR was analysed by quantitative immunofluorescence at 1 hour post IR when enoxacin-stimulated DDR signalling was maximum for all the factors tested (Figs 1 and 2A,B). While removal of TRBP or PACT alone was not sufficient to impair DDR, we observed a slight reduction of DDR signalling when the two DICER co-factors were depleted together (Fig. S3), suggesting that both proteins may contribute to DICER functions in regulating DDR. Coherent with previous findings^6,26^, the simultaneous depletion of all three GW182 proteins (TNRC6A, B and C), which are required for miRNA-dependent post-transcriptional gene silencing (PTGS), had no impact on DDR activation (Fig. S3A,C). Nonetheless, we observed that TRBP loss completely prevented the ability of enoxacin to enhance DDR foci intensity (Fig. 3). Importantly, the knockdown of PACT, which is not targeted by enoxacin, as well as GW182 removal did not affect the ability of enoxacin to enforce the DDR (Fig. 3).

Overall, these data demonstrate that the effects of enoxacin on the DDR are specific for TRBP activity and, notably, that are not caused by miRNA-mediated PTGS.

### Enoxacin stimulates generation of DDRNAs that guide 53BP1 recruitment to DSBs

**Figure 4:**
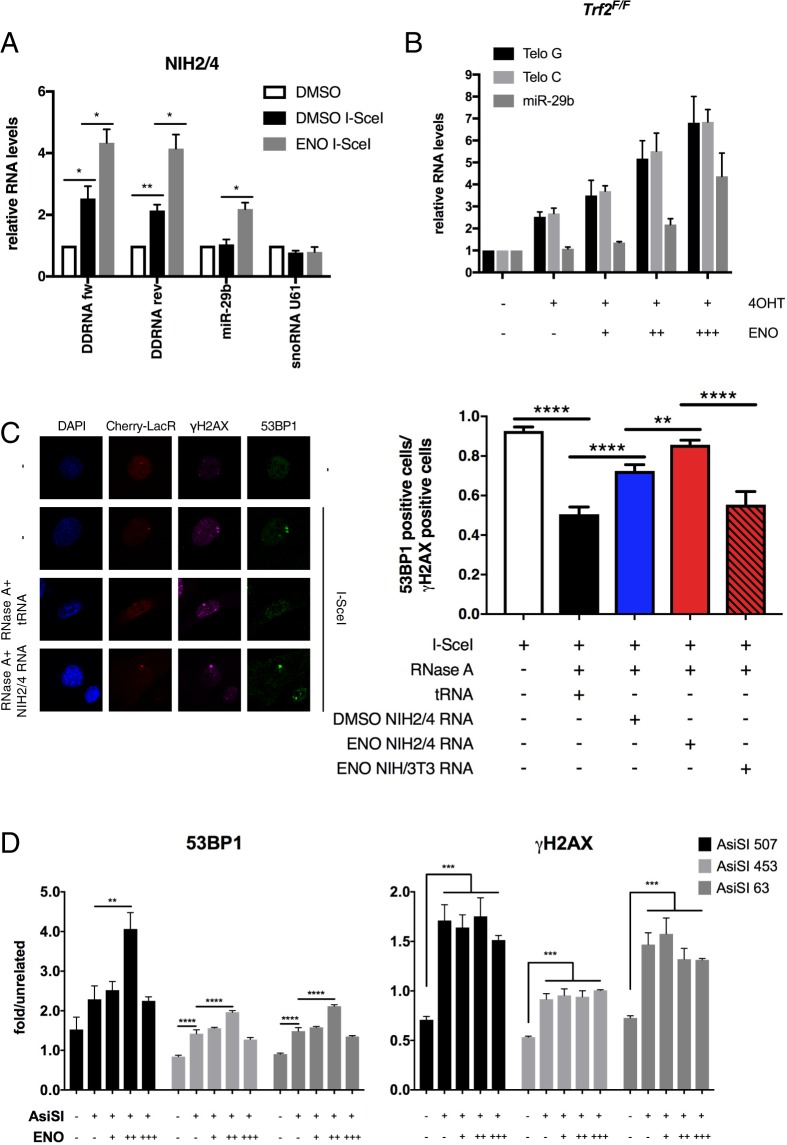
Enoxacin stimulates DDRNA generation and 53BP1 recruitment to DSB. (**A**) DDRNA profiling was performed by strand-specific qRT-PCR on gel-extracted RNAs from NIH2/4 cells expressing or not I-SceI and treated with 150 µM enoxacin (grey bars) or with DMSO (white and black bars) for 24 hours. DDRNA levels were measured with distinct primers for each strand (DDRNA fw and DDRNA rev). As a positive control, miR-29b expression was analysed in parallel; levels of an unrelated small RNA (snoRNA U61) were also studied. RNA levels are relative to untreated uncut samples (white bars); values are the means ± s.e.m. from three independent experiments. (**B**) qRT-PCR analysis of tDDRNA expression on gel-extracted RNAs from *Trf2*^*F/F*^ MEFs treated with 4OHT along with 50 (+ENO), 100 (++ENO) or 200 µM (+++ENO) enoxacin for 48 hours. tDDRNAs bearing the telomeric G-rich (Telo G) or C-rich (Telo C) strand sequence were profiled along with an enoxacin-responsive miRNA (miR-29b) as a positive control. RNA levels were normalised on synthetic cel-miR-67* used as a spike-in and shown as relative to untreated cells. Values are the means ± s.e.m. from three independent experiments. (**C**) Left panels: representative confocal-images showing γH2AX (magenta) and 53BP1 (green) focus formation at I-SceI cut sites, marked by cherry-LacR expression (red); nuclei were counter-stained with DAPI (blue). Following RNase A treatment, cut NIH2/4 cells were incubated with total RNA from I-SceI expressing NIH2/4 cells treated with enoxacin (ENO NIH2/4 RNA) or with DMSO only (DMSO NIH2/4 RNA), or with yeast tRNA as a control. Total RNA from enoxacin-treated parental cells (ENO NIH/3T3 RNA) was also used as a negative control. The histograms show the fraction of 53BP1-positive cells relative to γH2AX-positive cells at the locus studied; values are the means ± s.e.m. from three independent experiments; 56 to 223 cells were scored for each sample. (**D**) AsiSI-ER U2OS cells were incubated with 50 (+ENO), 100 (++ENO) or 200 µM (+++ENO) enoxacin for 24 hours prior to treatment with 300 nM 4OHT to induce AsiSI cleavage (+AsiSI). 4 hours post 4OHT administration, cells were analysed by ChIP. Histograms show 53BP1 (left panels) and γH2AX (right panels) occupancy at selected AsiSI cut sites. Values are normalised to an unrelated locus and represent the means ± s.e.m. of three technical replicates from two independent experiments.

We previously demonstrated that the recruitment of DDR factors to sites of damage is promoted by the administration of DDRNAs^6,8^, thus we explored whether the observed increased DDR signalling induced by enoxacin was the result of the augmented levels of DDRNAs generated at DSBs. To do so, we profiled DDRNA expression after administrating enoxacin to NIH2/4 murine fibroblasts, in which a single DSB can be induced at a specific and traceable locus, containing an I-SceI recognition site flanked by Lac-operator sequences, by the ectopic expression of I-SceI meganuclease^33^ - this cell line has been previously characterized for the induction of DDRNAs^6,8^. NIH2/4 cells expressing an inducible version of I-SceI were treated with enoxacin, or its vehicle DMSO, before DSB induction. Short RNAs (<30 nt) were cell extracted and size selected by gel-extraction prior to strand-specific quantitative RT-PCR (qRT-PCR) analysis. As expected, I-SceI-induced DSB was accompanied by an increase in the levels of both strands of DDRNAs originating at the locus and bearing Lac sequences (Fig. 4A). Most importantly, enoxacin treatment significantly enhanced the expression of DDRNAs by a fold-induction similar to the one we observed for miR-29b, a miRNA previously reported to respond to enoxacin treatment^18,23^, whereas an unrelated RNA (snoRNA U61) remained unaffected (Fig. 4A). To extend our conclusions to human cells and to evaluate the effect of TRBP loss on enoxacin-induced DDRNA production, we transfected I-HeLa111 cells, which carry the same I-SceI recognition site integrated in NIH2/4 cells flanked by Lac sequences and express the inducible I-SceI^34^, with siRNAs against human TRBP before cut induction and enoxacin administration. Consistent with what observed in the NIH2/4 cell line (Fig. 4A), enoxacin treatment elevates the levels of DDRNAs originated at the locus also in this human cellular system (Fig. S4A). Furthermore, knock down of TRBP in I-SceI cut untreated cells partially decreased DDRNA levels (Fig. S4A). Most importantly, enoxacin-mediated induction of DDRNAs was abrogated by TRBP depletion (Fig. S4A).

The latter results demonstrated the impact of enoxacin on DDRNAs generated at an engineered locus. To test if enoxacin could enhance the accumulation of DDRNAs produced at endogenous genomic sites, we profiled the expression of telomeric DDRNAs (tDDRNAs), which we have recently demonstrated to be produced at dysfunctional telomeres lacking the shelterin component Trf2^7^, by qRT-PCR following enoxacin administration. Thus, we treated inducible Trf2 knock-out mouse embryonic fibroblasts, *Trf2*^*F/F*^ MEFs^35^, with 4-hydroxytamoxifen (4OHT) to induce Trf2 loss and thus telomere uncapping along with increasing enoxacin concentrations. Excitingly, we observed that enoxacin increases tDDRNA production at uncapped telomeres and in a dose-dependent manner (Fig. 4B).

We next assessed if such increased levels of DDRNAs observed following enoxacin treatment could be functionally relevant for DDR activation in cells. We previously demonstrated that in NIH2/4 cut cells 53BP1 focal accumulation was impaired by transient RNase A treatment and it was rescued by subsequent incubation with total RNA purified from cut cells, but not from parental cells lacking the genomic cut locus, or with synthetic locus-specific DDRNAs – thus in a miRNA-independent manner^6^. We took advantage of this system to test the ability of RNA derived from enoxacin-treated cells in fostering 53BP1 focus reconstitution after RNase A treatment. To this aim, I-SceI expressing NIH2/4 cells were gently permeabilized and subjected to a brief RNase A treatment and then incubated with equal amounts of total RNA extracted from cut NIH2/4 cells treated with enoxacin or DMSO (as in Fig. 4A), or with tRNA as control. As expected, 53BP1 accumulation to the site of DSB, marked by the cherry-LacR protein that binds the Lac-operator repeats flanking I-SceI restriction site, was reduced by RNase A treatment and it was rescued by the addition of total RNA extracted from DMSO-treated cut cells (Fig. 4C). Remarkably, total RNA extracted from enoxacin-treated cells was significantly more effective at restoring 53BP1 focal accumulation to the DSB (Figs 4C and S4B). Notably, 53BP1 localization at DSB was not recovered when total RNA from an enoxacin-treated parental cell line (ENO NIH/3T3 RNA, Fig. 4C), not containing the I-SceI target site, was used for complementation. We confirmed that enoxacin treatment had the same efficiency in both NIH/3T3 and NIH2/4 cell lines by probing for miR-29b levels (Fig. S4C). Since NIH2/4 and NIH3T3 share the same genome and likely the same cellular RNA except for the RNA generated at the integrated locus, our data indicate that no other RNAs that might be boosted by enoxacin control 53BP1 recruitment to damaged sites other than those originating at the DSB.

To further confirm the effects of the augmented production of DDRNAs on 53BP1 recruitment to damaged sites, we treated U2OS cells expressing an inducible AsiSI endonuclease (AsiSI-ER)^36^ with increasing amounts of enoxacin prior to AsiSI induction and monitored 53BP1 occupancy to chromosomal endogenous DSBs by Chromatin Immuno-Precipitation (ChIP). AsiSI cut efficiency among samples was evaluated by probing for γH2AX at selected AsiSI restriction sites. We observed that 53BP1 occupancy at these loci increased upon DSB induction and it was further boosted by 100 μM enoxacin while γH2AX levels were unaffected (Fig. 4D), thus mirroring what already observed by immunofluorescence studies (Fig. S1). To further substantiate the specificity of enoxacin effects on TRBP functions, we knocked down TRBP in AsiSI-ER U2OS cells before enoxacin treatment and AsiSI cut induction and evaluated 53BP1 binding to individual DSBs. Consistently to what observed by immunofluorescence analyses (Fig. 3), 53BP1 accumulation at all tested loci was not elevated in enoxacin-treated cells lacking TRBP (Fig. S4D).

Together, these results indicate that enoxacin stimulates the generation of DDRNAs at chromosomal DSBs and at dysfunctional telomeres, and that this enforces 53BP1 recruitment to DNA lesions independently from miRNA-mediated effects.

### Enoxacin improves 53BP1-dependent DNA damage repair

**Figure 5:**
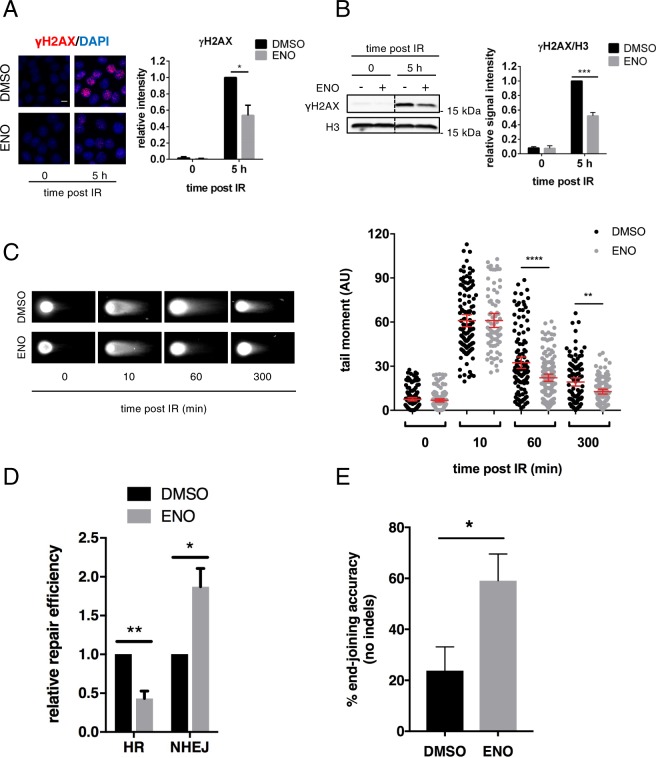
Enoxacin enhances DNA repair through accurate NHEJ. (**A**) HeLa cells treated as in Fig. 1 were fixed 5 hours post IR and immuno-stained for γH2AX (red); nuclei were counter-stained with DAPI (blue). Scale bar = 10 µm. Histograms show the intensity of γH2AX foci per nucleus relative to untreated irradiated cells (5 h, black bar). Values are the means ± s.e.m. of three independent experiments (Student’s t-test). (**B**) Whole protein lysates from HeLa cells treated as in (**A**) were probed for γH2AX by western blot. Dashed lines indicate the position where the blot was cropped. A full-length version of the same blot is provided in Fig. 5SA. Histograms show γH2AX levels relative to untreated irradiated cells (5 h, black bar). H3 was used as a loading control. Values are the means ± s.e.m. of three independent experiments (Student’s t-test). (**C**) Left panels: representative images of neutral comet assay performed in HeLa cells treated and irradiated as in Fig. 1. The dot plot on the right shows the quantification of IR-induced DNA damage by tail moment analysis. Red bars indicate the average values ±95% CI from at least three independent experiments; at least 100 cells per sample were scored. Time 0 post IR refers to not irradiated cells. (**D**) DR-GFP (HR) or EJ5-GFP (NHEJ) U2OS cell lines were treated with (ENO) or without enoxacin (DMSO) along with I-SceI expression. DSB re-joining events were evaluated by qPCR with primers spanning I-SceI cut sites performed on gDNA collected 72 hours after I-SceI expression. *β-ACTIN* gene DNA was used as a normaliser. Repair efficiency is shown relative to DMSO-treated cells and represented as the means ± s.e.m. of three independent experiments (Student’s t-test). (**E**) Re-joined fragments from EJ5-GFP U2OS gDNA described in (**D**) were amplified by PCR, cloned and analysed by Sanger sequencing for the presence of indels at the I-SceI site. The histogram shows the percentage of re-joining events containing no indels from DMSO or enoxacin (ENO) treated cells. Values are the averages ± s.e.m. of three independent experiments. At least 20 clones for sample were analysed.

DICER ablation was shown to cause altered DNA damage repair and genome instability^14–16^. Therefore, we hypothesised that enoxacin administration could not only enhance DDR signalling but also improve DNA damage repair by boosting DICER activity. Thus, we treated HeLa cells with either enoxacin or DMSO as a control and analysed γH2AX levels 5 hours post IR to monitor their disappearance as readout of DNA repair. While enoxacin administration did not alter γH2AX levels at early time points (Fig. 1), it significantly reduced γH2AX at later time points, as independently assessed by immunofluorescence and western blot analyses (Figs 5A,B and S5A), suggesting improved DNA repair despite equal amounts of DNA damage inflicted. To directly evaluate the effect of enoxacin on DNA damage repair efficacy, we monitored the levels of DSBs by performing a neutral comet assay in HeLa cells treated or not with the drug prior to IR. We observed a significant reduction in comet tail length at 1 and 5 hours (60, 300 min) post IR in enoxacin-treated samples, despite unaltered comet tail length right after IR (10 min, Fig. 5C). Most importantly, while TRBP loss abrogated enoxacin-mediated comet tail reduction, PACT knockdown only attenuated such an effect (Fig. S5B). Taken together, these results indicate that enoxacin administration increases the efficiency of DNA repair by acting mainly in a TRBP-dependent manner.

DSBs can be repaired either by NHEJ or HR^37^. To evaluate the impact of enoxacin on each of these major repair pathways, we administrated it to DR-GFP and EJ5-GFP U2OS cell lines, two well-established cellular systems to study DNA repair by HR and NHEJ, respectively^38,39^. These cell lines have an integrated GFP-based reporter construct bearing I-SceI recognition sites. After I-SceI-mediated DSB induction, repair either by HR (DR-GFP) or by NHEJ (EJ5-GFP) can be monitored. To directly study DNA repair events at these loci, we performed qPCR on genomic DNA with primers spanning I-SceI cut sites^38,40^. We observed that, while DNA damage repair through NHEJ was significantly increased in enoxacin-treated cells, HR-efficiency was reduced (Figs 5D and S5C,D). Enoxacin did not have an impact on cell-cycle distribution in either cellular systems as evaluated by flow cytometry (Fig. S5E). These results indicate that enoxacin-administration leads to a faster DNA repair by promoting NHEJ at the expenses of HR.

Differently from HR, NHEJ is generally considered as an error-prone repair process that may lead to nucleotide mis-incorporation (insertions) or loss (deletions) at re-joined DSBs^41^. To test if enoxacin impacted not only on the speed but also on the accuracy of NHEJ, genomic DNA extracted from untreated or enoxacin-treated EJ5-GFP U2OS cells transfected with I-SceI for 3 days (Fig. 5D) was amplified by PCR with primers spanning I-SceI cut sites. PCR products were gel purified (Fig. S5C), cloned and analysed by Sanger sequencing for insertions and deletions (indels) at the I-SceI junction. Strikingly, sequencing results indicate that accuracy of NHEJ in enoxacin-treated cells was significantly better (59.1 ± 10.5% accurately repaired clones) compared to control cells (23.8 ± 9.3%) (Fig. 5E). Moreover, indels length was reduced from 11.81 ± 2.61 bp in control samples to 3 ± 1.2 bp in enoxacin-treated cells (Fig. S5F). These results indicate that enoxacin administration reduces mutagenic repair by promoting accurate DNA end-joining.

**Figure 6:**
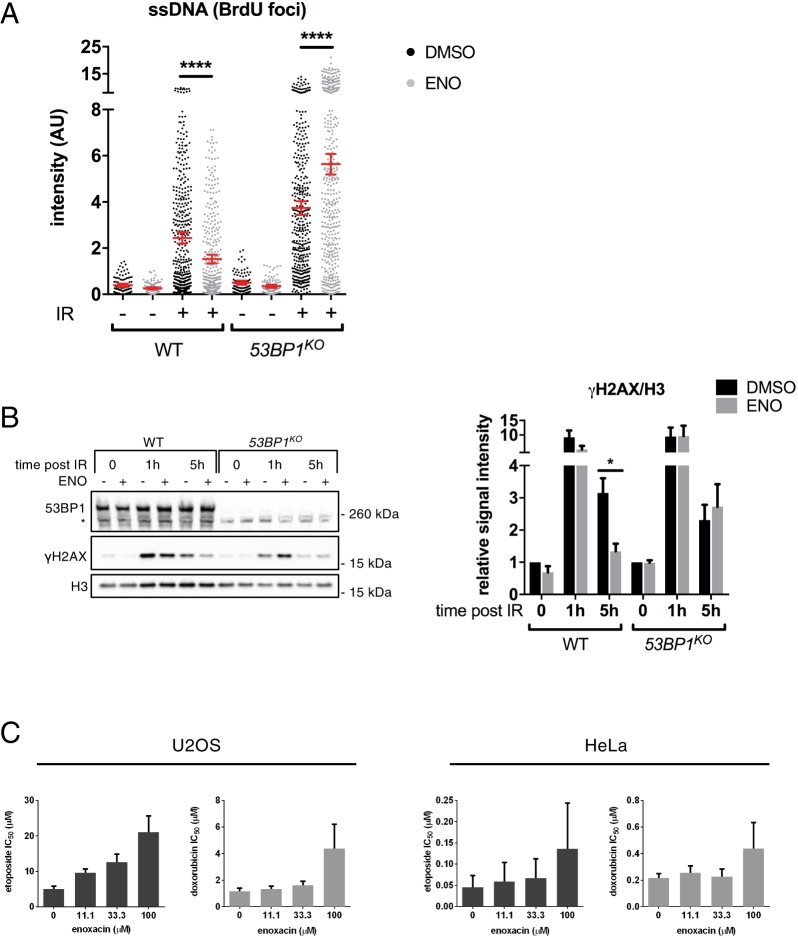
Enoxacin promotes NHEJ towards HR via 53BP1 and improves cell survival following chemotherapeutic agent administration. (**A**) DNA-end resection was assessed by monitoring ssDNA levels through BrdU native staining. Wild-type (WT) and *53BP1*^*KO*^ U2OS cells were treated with enoxacin (ENO) or DMSO for 48 hours before IR, fixed 5 hours after IR (IR+) and immuno-stained for BrdU. Not irradiated cells were also analysed (IR−). The plot shows the intensity of BrdU foci per nucleus. Red bars indicate the average values ±95% CI from three independent experiments; at least 300 cells per sample were scored. (**B**) Wild-type (WT) and *53BP1*^*KO*^ U2OS cells were incubated with (ENO+) or without (ENO−) enoxacin for 48 hours before IR. Total protein lysates were collected at the indicated time points post-irradiation and probed by immunoblotting for 53BP1 and γH2AX. H3 was used as a loading control. The asterisk marks unspecific signals. Histograms show γH2AX levels relative to H3; values are normalised on not-irradiated cells (0) treated with DMSO and shown as the averages ± s.e.m. of three independent experiments (Student’s t-test). (**C**) Enoxacin improves cancer cell survival following genotoxic treatment. U2OS and HeLa cells were treated with increasing doses of etoposide or doxorubicin alone or in the presence of different concentrations of enoxacin for 24 h. The impact of the drug treatments on cell viability was assessed through a real-time survival assay and used to calculate chemotherapeutics’ IC_50_. Histograms show etoposide (dark grey bars) and doxorubicin (light grey bars) IC_50_ in response to increasing enoxacin concentration. Values are the means ±90% CI of three technical replicates.

53BP1, which associates with DDRNAs in a manner dependent on its Tudor domain^8^, is one of the major players in regulating the cell choice between NHEJ and HR pathways. In particular, it prevents DNA-end resection, a critical upstream step of HR, consequently inhibiting HR while promoting NHEJ^37,42^. Since enoxacin increases DDRNA levels and promotes 53BP1 accumulation at DSBs (Figs 4 and S4), we examined the impact of this drug on DNA-end resection in wild-type and CRISPR/Cas9D10A-mediated 53BP1 knockout (*53BP1*^*KO*^) U2OS cells^43^. To do so, we performed BrdU native staining to detect single-stranded DNA (ssDNA) generated at resected DSBs^44^. We observed a significant reduction in the intensity of BrdU signals in wild-type enoxacin-treated cells at 5 hours post IR (Fig. 6A), coherently with the observed reduced HR (Fig. 5D) and augmented 53BP1 (Figs 1, 2C,D, 3 and 4C,D). This effect was reversed in the absence of 53BP1, consistent with 53BP1-dependent hampered DNA resection (Fig. 6A). We corroborated these data by determining the levels of the phosphorylated replication protein A (pRPA^S4/8^), a marker of ongoing DNA resection (Fig. S6A,B). Moreover, enoxacin reduced the focal accumulation of BRCA1, a central DNA repair factor which promotes HR by antagonising 53BP1 functions^37^, while leaving unaltered its total amounts (Fig. S6C–E).

To confirm that the improved DNA repair observed after enoxacin-administration (Fig. 5) depended on 53BP1 activity, we monitored γH2AX levels in wild-type and *53BP1*^*KO*^ cells treated with enoxacin by immunoblotting at different time points post IR. As already observed in HeLa cells (Fig. 5), enoxacin treatment of wild-type U2OS significantly reduced γH2AX signal at 5 hours after IR, confirming the ability of this drug to improve DNA damage repair (Fig. 6B). Most excitingly, enoxacin administration to *53BP1*^*KO*^ cells failed to reduce γH2AX levels at the same time point post IR (Fig. 6B). We further strengthened these findings by knocking down 53BP1 in EJ5-GFP (NHEJ) or DR-GFP (HR) U2OS cells prior to enoxacin administration and I-SceI expression. Consistent with previous reports^45^, we observed that 53BP1 depletion slightly reduced NHEJ efficiency and, most importantly, that cells lacking 53BP1 failed to increase both NHEJ efficiency and accuracy when treated with enoxacin (Fig. S6F,G and I). Also, following TRBP knockdown, enoxacin administration did not improve NHEJ repair (Fig. S6G), confirming the specificity of enoxacin for TRBP activity. In addition, 53BP1 removal attenuated the inhibitory effect of enoxacin on HR (Fig. S6H).

As NHEJ is particularly relevant in non-proliferating cells and given the reported positive impact of enoxacin in an *in vivo* mouse model of ALS^24^ consistent with an emerging role of DNA damage in neurodegeneration^46,47^, we tested the effect of enoxacin on DNA repair in post-mitotic neurons. Excitingly, we observed that primary cortical mouse neurons treated in culture with increasing enoxacin concentrations displayed a dose-dependent reduction of residual γH2AX signals at 1 and 5 hours post IR as compared to DMSO-treated neurons, despite equal initial levels, thus indicating increased DNA repair efficiency (Fig. S6J,K).

Collectively, our results indicate that enoxacin promotes DNA repair, including in primary neurons, through accurate NHEJ by increasing 53BP1 recruitment to DSBs.

### Enoxacin rescues cell survival after DNA damage induction

Enoxacin has been described to inhibit the growth of human cancer cell lines^18,23,48,49^. However, as we have shown that enoxacin improves DNA damage resolution (Fig. 5), we reasoned that it could also favour the survival of damaged cells. Thus, we tested the impact of enoxacin on the growth of cancer cells in the presence of DNA damaging chemotherapeutic drugs. We incubated U2OS and HeLa cells with increasing amounts of etoposide or doxorubicin, two compounds that generate DSBs^50,51^, along with different concentrations of enoxacin and monitored cell viability through a real-time survival assay. We observed that enoxacin treatment enhanced damaged cell survival in a dose-dependent manner, causing an increase of chemotherapeutics’ half maximal inhibitory concentration (IC_50_) (Figs 6C and S6L). These findings indicate that enoxacin may be detrimental in cancer therapy settings by reducing the impact of chemotherapeutic treatments through improved DNA damage repair and cell survival.

## Discussion

The DDR is a fundamental sequence of events that protect cells from genomic instability by guiding the faithful repair of damaged DNA. Alterations affecting DDR and DNA repair genes are considered to be causally related to the onset and progression of cancer, ageing, neurodegenerative pathologies and other diseases^1,52,53^.

Recently, a new family of DROSHA- and DICER-dependent sncRNA was found to play important roles in DDR signalling and DNA repair^12,13,54^, providing new insights into the molecular mechanisms controlling such processes and a novel therapeutic target for the treatment of DDR/DNA repair-related defects. Indeed, we have recently demonstrated that impairing the function of DDRNAs and their precursors by antisense oligonucleotides reduces DDR activation and DNA repair of DSBs^8^.

Here, by promoting the synthesis of DDRNAs with a pharmacological treatment we were able for the first time to positively modulate DDR activation and DNA repair. We took advantage of the small molecule enoxacin, recently found to improve the endoribonuclease activity of the DICER-complex, by facilitating the interaction of TRBP to the RNA substrates^17,18^. Specifically, we discovered that enoxacin treatment of cultured cells exposed to DNA damage strongly increases DDR activation (Figs 1 and 2). Importantly, we demonstrated that this effect, while relying on TRBP activity, does not require the functions of the GW182 protein family, necessary effectors for miRNA-guided gene-silencing (Fig. 3). These results indicate that the canonical functions of miRNAs are dispensable for enoxacin-mediated DDR enhancement and suggest an engagement of non-miRNA DICER-products. Indeed, we found that enoxacin-exposure increases the production of DDRNAs generated at chromosomal DSBs as well as at dysfunctional telomeres in a dose-dependent manner, and that they are functionally relevant for DDR activation, specifically by favouring 53BP1 recruitment to damaged sites (Figs 4 and S4). Most importantly, in an experimental setup in which miRNAs cannot play a significant role, we showed that 53BP1 accumulation at DSBs in RNA-depleted cells was more efficiently restored by supplying RNA purified from enoxacin-treated cells than from control untreated cells (Fig. 4C). In addition, RNA extracted from enoxacin-treated cells not containing the target site, in which DSB was induced and DDRNA were generated, was not able to complement 53BP1 foci loss (Fig. 4C). This supports a model in which enoxacin-enhanced DDR activation is mediated by DDRNAs originated at DSB and much less by other RNAs generated elsewhere in the genome.

A recent report showed that embryonic stem cells and neural precursor cells lacking DICER displayed marked genomic instability and increased cell death^16^, uncovering an essential role of DICER in DNA damage repair *in vivo*. Here, we demonstrated that enoxacin administration is indeed beneficial for DNA damage repair by NHEJ. Human cells treated with enoxacin before exposure to IR showed the ability to accelerate DNA damage resolution (Fig. 5). Interestingly, while such an effect disappears if TRBP is depleted through RNAi, it is attenuated upon PACT knock-down (Fig. 5A). These findings may be explained by the partial redundancy of TRBP and PACT functions in supporting DICER activity^55^, thus they may share roles also in DDR (Fig. S3C). More importantly, the ability of enoxacin to accelerate DNA repair is lost in the absence of 53BP1 (Fig. 6B). This is consistent with the previously-reported role of DICER products in controlling 53BP1 accumulation in DDR foci^6,14^, likely by their association with the protein through its Tudor domain^8,56^. In addition, this substantiates the independency of such mechanism from miRNA-mediated PTGS.

53BP1 not only favours NHEJ-mediated repair through the inhibition of DNA-end resection and the consequent impairment of HR^42^, but it also ensures DNA end-joining accuracy by limiting the impact of nucleotide incorporation/loss at damaged sites^57^. We indeed observed that enoxacin fuels NHEJ by hampering HR through 53BP1-dependent suppression of DNA-end resection (Figs 5D, 6A, S5C,D, S6A,B and G,H). These findings are supported by a recently proposed role for DICER and DSB-associated sncRNAs in controlling NHEJ-mediated repair in mammals and in plants^7,9,58^. Furthermore, the effects of enoxacin observed here are not limited to improve the speed of DNA repair but also its accuracy, by reducing both the frequency and the length of indels at the re-joined sites (Figs 5E and S5F).

Importantly, we observed that enoxacin is effective at improving DNA damage resolution also in primary neurons (Fig. S6J,K). As mounting evidence have linked DNA repair defects to neuro-degeneration in ALS^46,47^, it is thus tempting to propose that the demonstrated beneficial effects of enoxacin in ALS^24^ could be the result of improved DNA damage repair in motor neurons.

Conversely, enoxacin was reported to be cytotoxic in a variety of tumour cell lines^18,23,48,49^. Our unanticipated results, showing that enoxacin actually improves cancer cells survival upon treatment with DNA damaging agents (Figs 6C and S6L), suggest a word of caution in the use of enoxacin in combination with standard DNA damaging treatments such as radiotherapy and chemotherapy in cancer therapy.

In conclusion, in this work we report the first pharmacological approach that potentiates DDR activation and DNA repair by promoting the synthesis of DDRNAs. In addition, our results also suggest a potential additional mechanism of action for the observed beneficial effects of enoxacin in neurodegenerative disorders.

## Materials and Methods

### Cell Culture

HeLa cells (ATCC) were cultured in MEM-GlutaMAX™ (Gibco) supplemented with 10% FBS, 1% NEAA, 1% Sodium Pyruvate and 1% penicillin/streptomycin (P/S). NIH/3T3 (ATCC) and NIH2/4 cells^33^ were grown in DMEM (Lonza) supplemented with 10% Tet System Approved FBS, 1% L-glutamine and 1% P/S; 400 µg ml^−1^ Hygromycin was added to the medium for culturing NIH2/4 cells. I-HeLa111 cells were cultured as described^34^. AsiSI-ER^36^, DR-GFP^39^, EJ5-GFP^38^, *53BP1*^*KO*^^43^, GFP-53BP1^32^, GFP-NBS1^31^ U2OS cell lines and normal U2OS cells (ATCC) were cultured in DMEM (Lonza) supplemented with 10% FBS, 1% L-glutamine and 1% P/S. *Trf2*^*F/F*^ mouse embryonic fibroblasts (MEFs) were cultured as described before^35^. All cell lines were tested for the presence of mycoplasma and found to be negative. All cells were maintained at 37 °C in a 5% CO_2_ humidified incubator. Primary cultures of mouse cortical neurons established from postnatal day 0 pups were plated on poly-L-lysine-coated dishes. Cultures were used for experiments after 14 days *in vitro* (DIV) and incubated at 37 °C during all treatments in Neurobasal Medium (Gibco) with B27 supplement.

### Cell treatments and transfection

For most of the experiments, enoxacin (E3764, Sigma-Aldrich) was dissolved in 100% DMSO and added to the growth medium at the final concentration of 50 µM. Generally, cells were grown in the presence of 50 µM enoxacin for 48 hours, unless stated otherwise. Cells were exposed to 2 or 5 Gy of ionizing radiation with a high-voltage X-ray generator tube (Faxitron X-Ray Corporation). siRNAs for human TARBP2, PACT, TNRC6A, -B and -C, 53BP1, and GFP or non-targeting siRNAs as negative controls, were purchased from Dharmacon (Table S1) and transfected using Lipofectamine® RNAiMAX (Thermo-Fisher) at the final concentration of 20 nM. Cherry-LacR and I-SceI expressing plasmids^33^ were transfected with Lipofectamine® 2000 (Thermo-Fisher). I-SceI expression in I-HeLa111 was induced by administrating 1 µg ml^−1^ doxycycline for 24 hours. To induce Trf2 knock-out, *Trf2*^*F/F*^ MEFs^35^ were incubated with 600 nM 4-hydroxytamoxifen (4OHT) for 48 hours.

### Immunofluorescence microscopy and quantitative analysis of DDR foci

For the immunofluorescence assays, cells were grown on coverslips, fixed with 4% paraformaldehyde (PFA) or methanol:acetone (1:1) and probed with the primary antibodies (Table S2) for 1 h at r.t. in 1X PBS supplemented with 0.5% (g/ml) BSA and 0.2% (ml/ml) fish gelatin (G7765, Sigma-Aldrich). After washing, cells were incubated with secondary antibodies for 45 min at r.t. in the same buffer. Cells were then washed thoroughly, stained with DAPI and mounted with Mowiol 4–88 (81381, Sigma-Aldrich). Images were acquired with wide-field Olympus Biosystems Microscope and MetaVue software. The intensity of DDR foci per nucleus was measured using CellProfiler software^59^. Briefly, images were first masked over DAPI-staining to select for nuclei and discard unspecific cytoplasmic signal. Next, foci were identified with the “enhance speckles” module and background signal filtered out using the manual thresholding strategy. The intensity of nuclear DDR foci was then calculated by multiplying the number of nuclear foci by their average intensity in each nucleus.

### Protein extraction and immunoblotting

Proteins were extracted with Laemmli buffer and fractionated onto a 4–15% and 4–12% SDS-PAGE (Bio-Rad, Thermo-Fisher). Proteins were then transferred onto nitrocellulose membrane (GE Healthcare) and probed over-night with primary antibodies (Table S2). Blot imaging was performed with ChemiDoc™ Imaging Systems (Bio-Rad) and quantification of protein levels was conducted by densitometric analysis with Image Lab™ software (Bio-Rad).

### Laser-induced DNA damage

Laser-induced DNA damage and live cell imaging were performed on a Leica TCS SP5 point scanning confocal microscope equipped with a Leica HCX PL APO 63X/1.4NA oil immersion and an environmental microscope incubator (OKOLab) set to 37 °C and 5% CO2 perfusion. The Leica TCS SP5 confocal microscope was driven by Leica LAS AF software. Cells were cultured in glass bottom dishes (Mattek P35G-1.5-14-C) and pre-sensitized for 72 h in 10 µm BrdU (Sigma-Aldrich). Laser micro-irradiation was carried out using a 50 mW 405 nm diode laser with a 100% power output. At 2X digital magnification, multiple regions of interest (ROI) were selected in each nucleus and the 405 nm laser was used to scan the ROIs for 50 iterations (total dwell time per pixel 490 microsec). GFP signal in laser-damage induced stripes were quantified by ImageJ by drawing the ROI of laser damage, accordingly to where ROI of laser damage was generated; the mean fluorescence intensity in each damaged area was measured and the mean intensity of an identical area in an undamaged region of the same nucleus was subtracted as background.

### RNA extraction and analysis

Total RNA was isolated using TRIzol Reagent (Thermo-Fisher). mRNA analysis was performed by quantitative RT-PCR (qRT-PCR) following DNaseI treatment (Thermo-Fisher) and retro-transcription with SuperScript® VILO cDNA Synthesis Kit (Thermo-Fisher). Small RNAs (<30 nt) were gel-purified after fractionation of total RNA along with 10 pg of synthetic *C. elegans* cel-miR-67* as a spike-in onto 10% Urea-PAGE and analysed using miScript II System (Qiagen) by qRT-PCR. Primers used for qRT-PCR analyses are listed in Table S1.

### RNase A treatment and complementation assay

RNA complementation experiments were conducted as described before^6^ with minor modifications. In brief, I-SceI- and Cherry-LacR-expressing NIH2/4 cells seeded on coverslips were permeabilized with 0.6% Tween-20 in PBS for 15 min at room temperature, with gentle agitation. RNase A (R5503, Sigma-Aldrich) was dissolved as already shown^60^ and added to permeabilized cells at the concentration of 1 mg ml^−1^ for 30 min at room temperature with agitation. After having washed thoroughly with cold PBS, RNase A was inactivated with RNaseOUT (Thermo-Fisher) in PBS supplemented with α-amanitin (A2263, Sigma-Aldrich) for 10 min at room temperature. Next, cells were incubated with 100 ng of total RNA from I-SceI expressing NIH2/4 treated with 150 µM enoxacin or DMSO for 24 h, or same amount of total RNA of enoxacin-treated NIH 3T3, or yeast tRNA as a control, in the presence of RNaseOUT and α-amanitin in PBS for 25 min at room temperature. Cells were then fixed with 4% paraformaldehyde prior to immuno-staining. Images were obtained with TCS SP2 AOBS confocal laser microscope (Leica) by sequential scanning. Cells positive for γH2AX presence at the Cherry-LacR spot were scored for 53BP1 signal.

### Chromatin Immuno-Precipitation (ChIP)

AsiSI-ER U2OS cells^36^ were treated with increasing enoxacin concentrations for 24 hours prior to induction of AsiSI nuclear translocation with 300 nM 4OHT. 4 hours post 4OHT administration, cells were cross-linked for 5.5 min at room temperature with Fixation Buffer (1% formaldehyde; 100 mM NaCl; 1 mM EDTA; 0.5 mM EGTA; 50 mM HEPES pH 7.4). Cross-linking was quenched by addition of glycine (125 mM). Cell pellets were first re-suspended in cold B1 Buffer (0.25% Triton X-100; 1 mM EDTA; 0.5 mM EGTA; 10 mM Tris pH 8; Proteases inhibitors (Roche); Microcystin (Enzo Life Sciences)), then in cold B2 Buffer (200 mM NaCl; 1 mM EDTA; 0.5 mM EGTA; 10 mM Tris pH 8; Proteases inhibitors; Microcystin), and finally in cold B3 Buffer (TE 1X; EGTA 0.5 mM). Pellets were sonicated using a Focused-Ultrasonicator Covaris (duty: 5.0; PIP: 140; cycles: 200; amplitude: 0; velocity: 0; dwell: 0; microTUBEs with AFA fiber). Sonicated chromatin was diluted in RIPA buffer (1% TritonX-100; 0,1% Na-Deoxycholate; 0,1% SDS; 64 mM NaCl; 10 mM Tris HCl pH 8.0) to give a concentration of approximately 100 μg in 400 μl per ChIP. After 2 h of pre-clearing at 4 °C with 20 μl of magnetic beads (Dynabeads® Protein G, Thermo-Fisher), samples were incubated overnight at 4 °C with specific antibodies (Table S2) or no antibody (mock). The bound material was recovered by 2 h incubation with 20 μl of magnetic beads per ChIP. Beads were then washed four times in RIPA buffer, once in LiCl buffer (250 mM LiCl; 0.5% NP-40; 0.5% Na Deoxycholate; 1 mM EDTA; 10 mM Tris-HCl pH 8) and finally in 1X TE. ChIPed material was eluted in 150 μl Elution Buffer (1% SDS; 10 mM EDTA; 50 mM Tris HCl pH 8) for 15 min at 65 °C. Samples were reverse-crosslinked by adding proteinase K (Thermo-Fisher) at 37 °C for 5 h and then at 65 °C overnight. DNA was purified with QIAquick PCR purification column (Qiagen) and analysed by qPCR with primers listed in Table S1. DDR factor occupancy at DSBs was studied on three different AsiSI genomic sites selected among the most effectively cut^61^.

### Comet assay

Neutral comet assay was performed following manufacturer’s instructions (Trevigen). Briefly, HeLa cells were trypsinized, washed once with ice-cold PBS and resuspended in cold PBS at the final concentration of 10^5^ cells ml^−1^. Cell suspension was then combined with pre-warmed low-melting agarose at a ratio of 1:10 (v/v) and poured onto the slides. Lysis was performed over-night at 4 °C. Electrophoresis was carried out in 1X Neutral Electrophoresis Buffer for 45 min at 21 V. After DNA precipitation and wash in 70% ethanol, slides were dried up and DNA stained with SYBR Gold (Thermo-Fisher) before epifluorescence microscopy analysis (Olympus Biosystems). Comet tail moment was calculated using OpenComet software^62^.

### HR and NHEJ repair assays

DR-GFP (HR) were treated with 5 µg ml^−1^ doxycycline to induce I-SceI expression, while EJ5-GFP (NHEJ) cells were transfected with I-SceI expressing plasmid or an empty vector as a control, and simultaneously incubated with 50 μM enoxacin (or DMSO). For RNAi experiments, these cells were transfected with 20 nM siRNAs against 53BP1 or with non-targeting siRNAs (Dharmacon, Table S1) for 48 h prior to enoxacin treatment and I-SceI expression. Genomic DNA (gDNA) was extracted with DNeasy Blood & Tissue Kit (Qiagen) at 24, 48 and 72 h after I-SceI expression. Efficiency of HR and NHEJ repair was measured by qPCR on gDNA with primers spanning I-SceI recognition sites (DRGFP F/R or EJ5GFP F/R for HR or NHEJ assay, respectively; Table S1). Primers for human β-ACTIN gene were used as a reference.

### Analysis of NHEJ accuracy

gDNA extracted from untreated or enoxacin-treated EJ5-GFP U2OS cells transfected with I-SceI for 3 days was amplified by PCR with Phusion® High-Fidelity DNA Polymerase (NEB) using primers encompassing I-SceI cut sites (EJ5GFP F/R). PCR products between 200–300 bp were gel purified, cloned into pCR™II-Blunt-TOPO^®^ vector (Thermo-Fisher) and analysed by Sanger sequencing for insertions and deletions (indels) at the I-SceI junction. 3 independent experiments for each sample were combined in the sequencing data.

### Cell cycle profiling

For cell cycle analysis, cells were fixed in 75% ethanol and stained with Propidium Iodide (PI) (Sigma-Aldrich, 50 µg ml^−1^) in 1X PBS supplemented with RNase A (Sigma-Aldrich, 250 µg ml^−1^). Samples were analysed on a BD Facs CantoII, using a 488 nm laser and 585/42 filter for PI. Acquisition was performed with the software BDFacsDIVA v6.1.1, and analysis was done using software ModfitLT3.0. At least 10^4^ events were analysed per sample.

### Native BrdU staining for ssDNA detection

Wild-type and *53BP1*^*KO*^ U2OS cells seeded on coverslips were incubated with 10 μM BrdU (Sigma-Aldrich) and simultaneously treated with 50 μM enoxacin or DMSO for 48 h before IR. 5 h after IR, cells were fixed in 4% PFA and stained with anti-BrdU antibody (GE Healthcare) for 1 h.

### Real-time survival assay

HeLa and U2OS cells (500/well) were seeded in 96-well plate in 50 µl of complete media and grown for 24 hours. Then, serial dilutions of doxorubicin and etoposide (Selleck Chemicals) were prepared in DMSO and each combined to enoxacin in complete media containing 2X RealTime-Glo reagents (Promega), such that each compound was at 2X the final concentration. 50 µl of drug combinations were dispensed on cells and luminescence was read after 24 hours on a Tecan Infinite F200 plate reader (at 37 °C). Half-maximal inhibitory concentration (IC_50_) was calculated using GraphPad Prism® software for the two chemotherapeutics in the absence or presence of increasing amounts of enoxacin. All experiments were performed in triplicate wells for each condition and repeated twice.

### Statistical Analysis

One-way or two-way ANOVA was used for multiple comparisons between samples, unless stated otherwise. *p < 0.05, **p < 0.01, ***p < 0.001, ****p < 0.0001.

### Use of mouse samples

All methods to obtain mouse primary neurons were carried out in accordance with relevant guidelines and regulations.

All experimental protocols were approved by the National Animal Care and Use Committee.

## Supplementary information

Supplementary Figures and Tables
