# Supplementary material for: Pharmacological boost of DNA damage response and repair by enhanced biogenesis of DNA damage response RNAs
Source: Sci Rep. 2019 Apr 23;9:6460. doi: 10.1038/s41598-019-42892-6 (PMC6478851; doi:10.1038/s41598-019-42892-6)
Supplement: Supplementary file 1 — Supplementary Figures and Tables [file 41598_2019_42892_MOESM1_ESM.pdf]

Supplementary Information

Pharmacological boost of DNA damage response and repair by enhanced biogenesis of DNA damage response RNAs

Ubaldo Gioia[1], Sofia Francia[1,2], Matteo Cabrini[2], Silvia Brambillasca[1], Flavia Michelini[1,#], Corey W. Jones-Weinert[1] and Fabrizio d'Adda di Fagagna[1,2]*

[1]IFOM – the FIRC Institute of Molecular Oncology, Via Adamello 16, 20139 Milan, Italy

[2]Istituto di Genetica Molecolare, Consiglio Nazionale delle Ricerche, 27100 Pavia, Italy

[#]Present address: Colonna (Rome), 00030, Italy

*To whom correspondence should be addressed. Tel: +39 02 574303 227; Fax: +39 02 574303 088; Email: fabrizio.dadda@ifom.eu

Figure S1

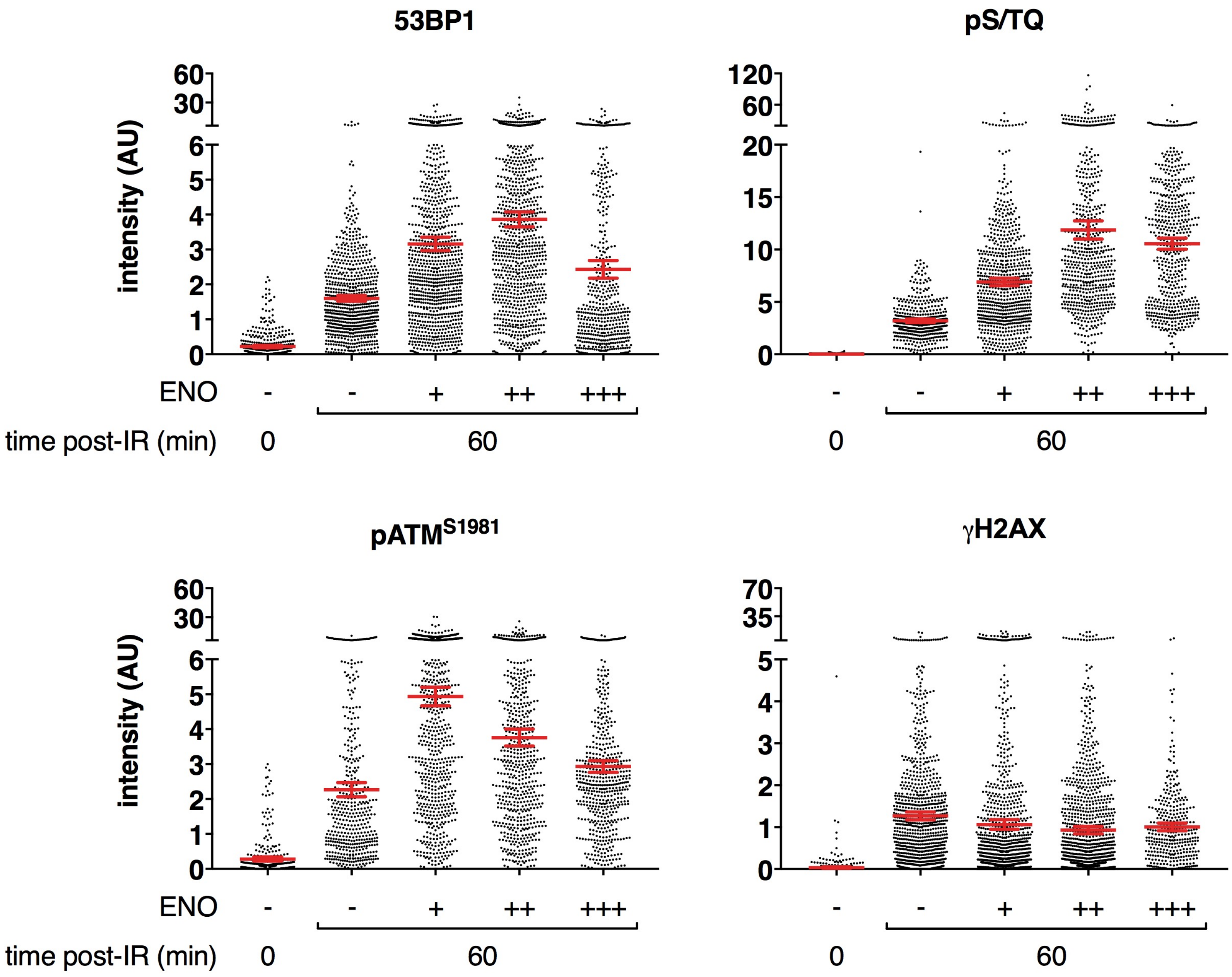

Figure S1. The dot plots show the intensity of DDR foci per nucleus of HeLa cells treated with 50 (+ENO), 100 (++ENO) or 200 μM (+++ENO) enoxacin for 24 hours prior to IR. Analysis of DDR activation was performed at 60 minutes post IR. Red bars represent the means ± 95% CI of three independent experiments; at least 300 cells per sample were scored.

Figure S2

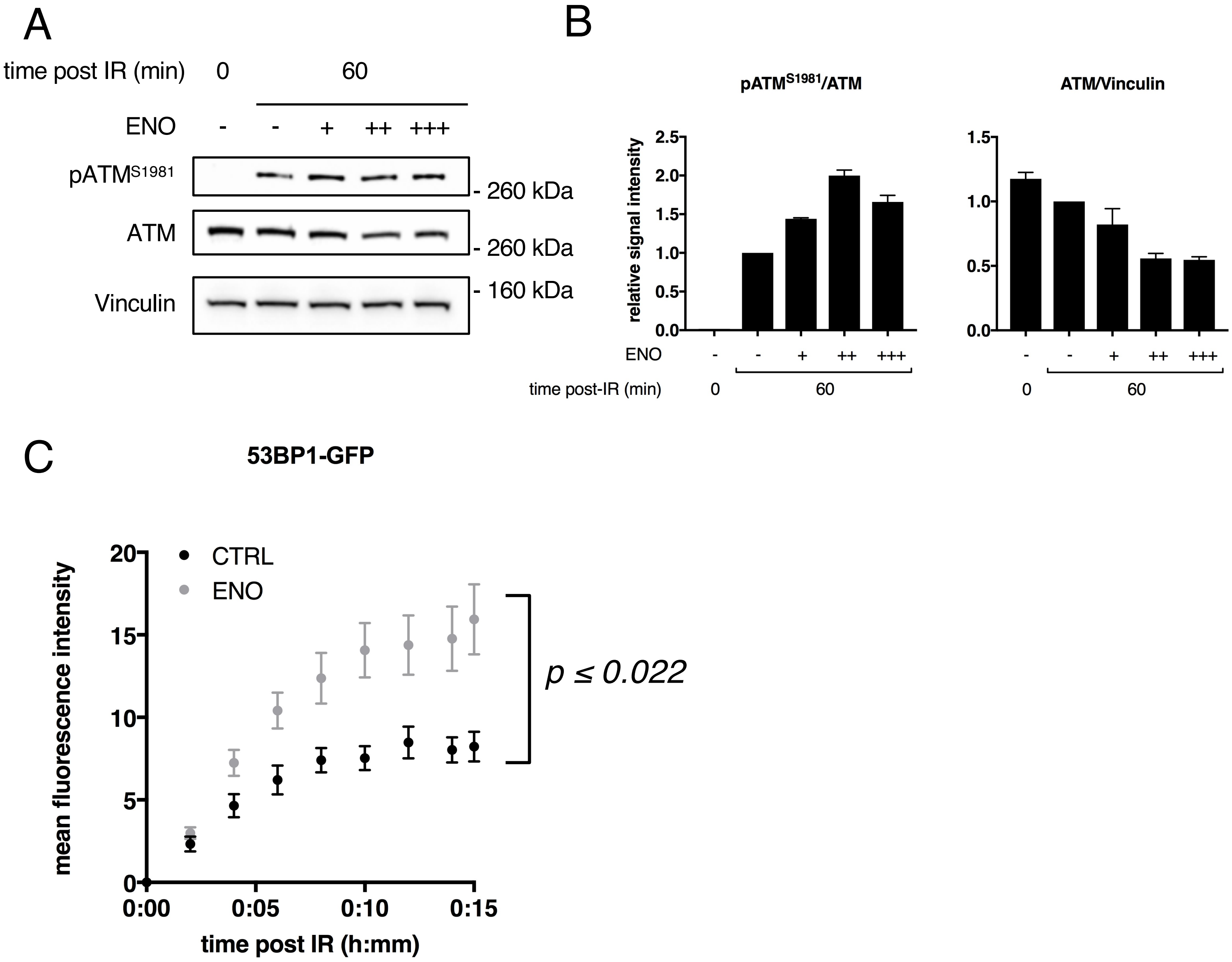

Figure S2. (**A**) Whole lysates from HeLa cells treated as in Figure S1 were probed for the indicated proteins by immunoblotting. (**B**) Histograms show the densitometric analysis of $pATM^{S1981}$ relative to total ATM and of total ATM relative to Vinculin. Values are the means ± s.e.m. of two independent experiments. (**C**) Analysis of 53BP1-GFP recruitment kinetics to stripes of micro-irradiation. Cells expressing 53BP1-GFP were treated as in Figure 2C. The dot plot shows 53BP1-GFP fluorescence intensity at laser-stripes measured over time post micro-irradiation (h:mm). Fluorescence intensity analysed before damage induction (time = 0) was set to 0. Values are the means ± s.e.m. (n=10). Time 0 post IR refers to not irradiated samples.

Figure S3

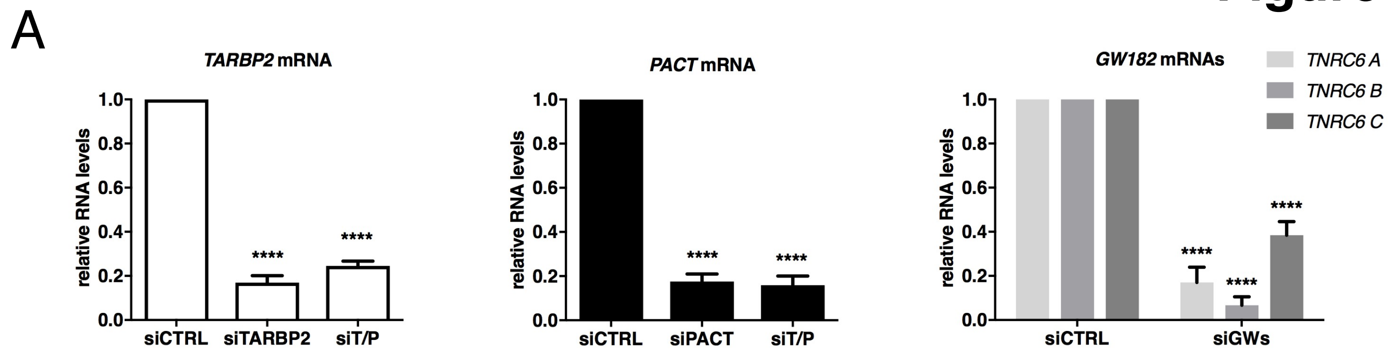

A
TARBP2 mRNA
PACT mRNA
GW182 mRNAs
TNRC6 A
TNRC6 B
TNRC6 C
relative RNA levels
siCTRL
siTARBP2
siT/P
siPACT
siGWs
****

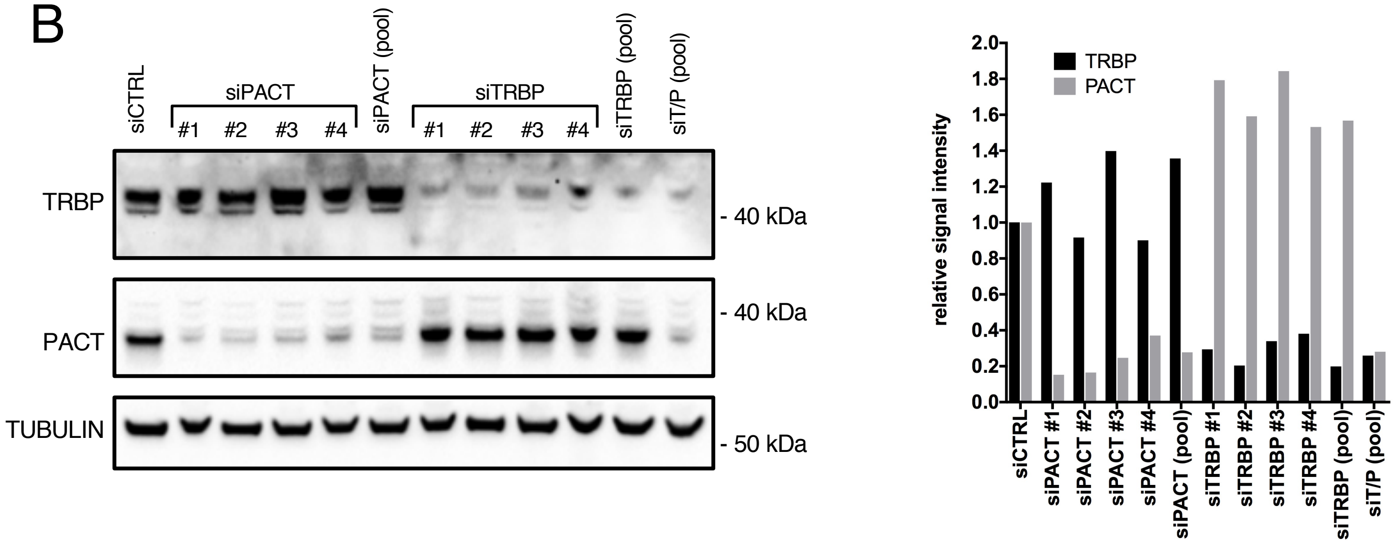

B
siCTRL
siPACT
#1
#2
#3
#4
siPACT (pool)
siTRBP
siTRBP (pool)
siT/P (pool)
TRBP
PACT
TUBULIN
- 40 kDa
- 40 kDa
- 50 kDa
relative signal intensity
siPACT #1
siPACT #2
siPACT #3
siPACT #4
siTRBP #1
siTRBP #2
siTRBP #3
siTRBP #4

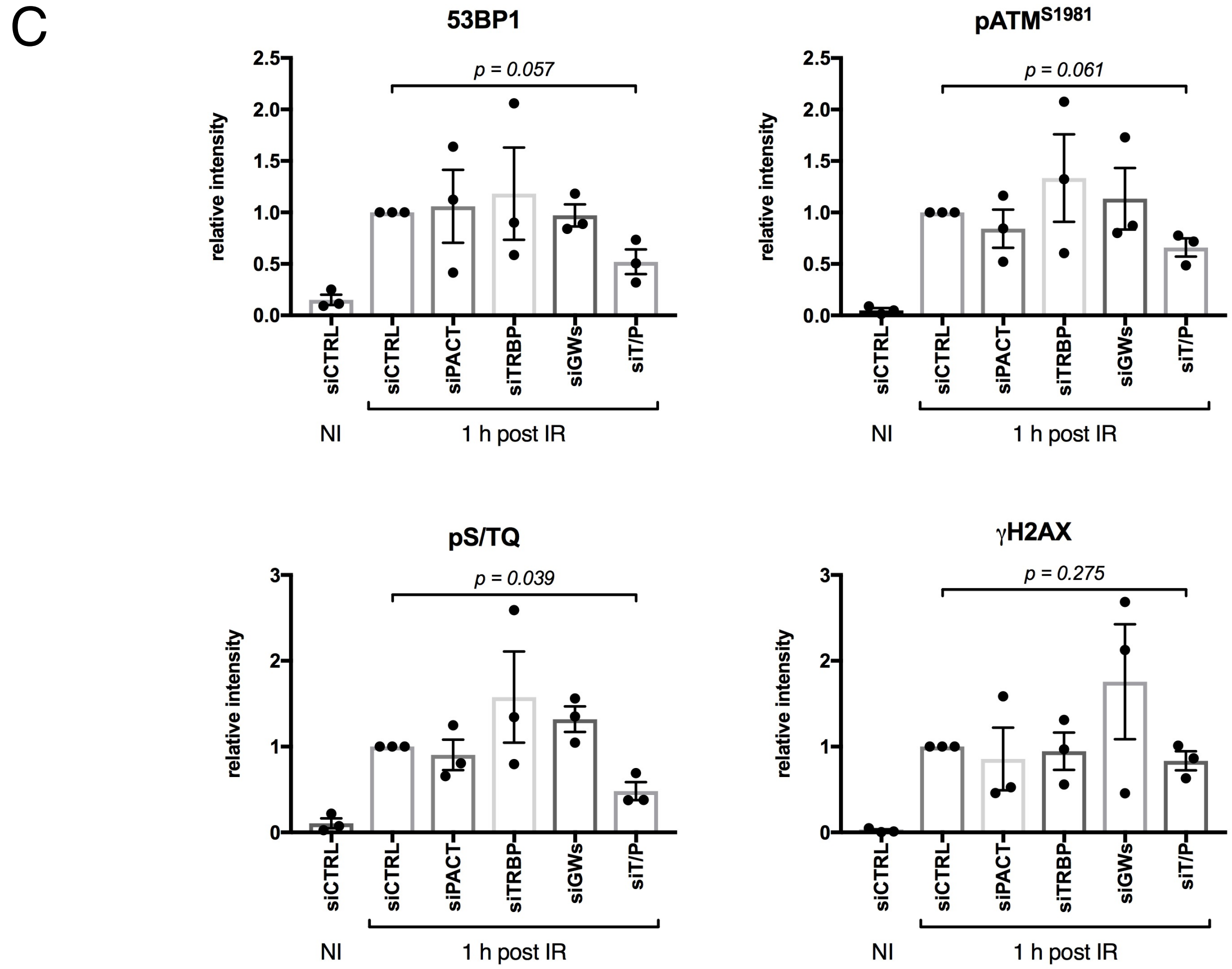

C
53BP1
pATM^S1981
pS/TQ
γH2AX
p = 0.057
p = 0.061
p = 0.039
p = 0.275
relative intensity
siCTRL
siPACT
siTRBP
siGWs
siT/P
NI
1 h post IR

Figure S3. (**A**) HeLa cells were transfected with siRNAs as in Figure 3. Total RNA was purified and analysed 48 hours after transfection. Histograms show the averages ± s.e.m. from at least three independent experiments (Student's t-test). (**B**) Whole protein lysates from HeLa cells transfected with the indicated siRNAs individually or as a pool, were probed for TRBP or PACT at 48 hours after transfection. Tubulin was used as a loading control. (**C**) HeLa cells were transfected with the indicated siRNAs 48 hours prior to IR. 1 hour post IR, cells were fixed and stained for 53BP1, pS/TQ, pATM^{S1981} or γH2AX. Histograms show the relative intensity of DDR foci; values are relative to irradiated cells transfected with a non-targeting siRNA (1 h post IR, siCTRL) and shown as the means ± s.e.m. of three independent experiments; at least 300 cells per sample were scored. siT/P = siTRBP + siPACT. NI = not irradiated samples.

Figure S4

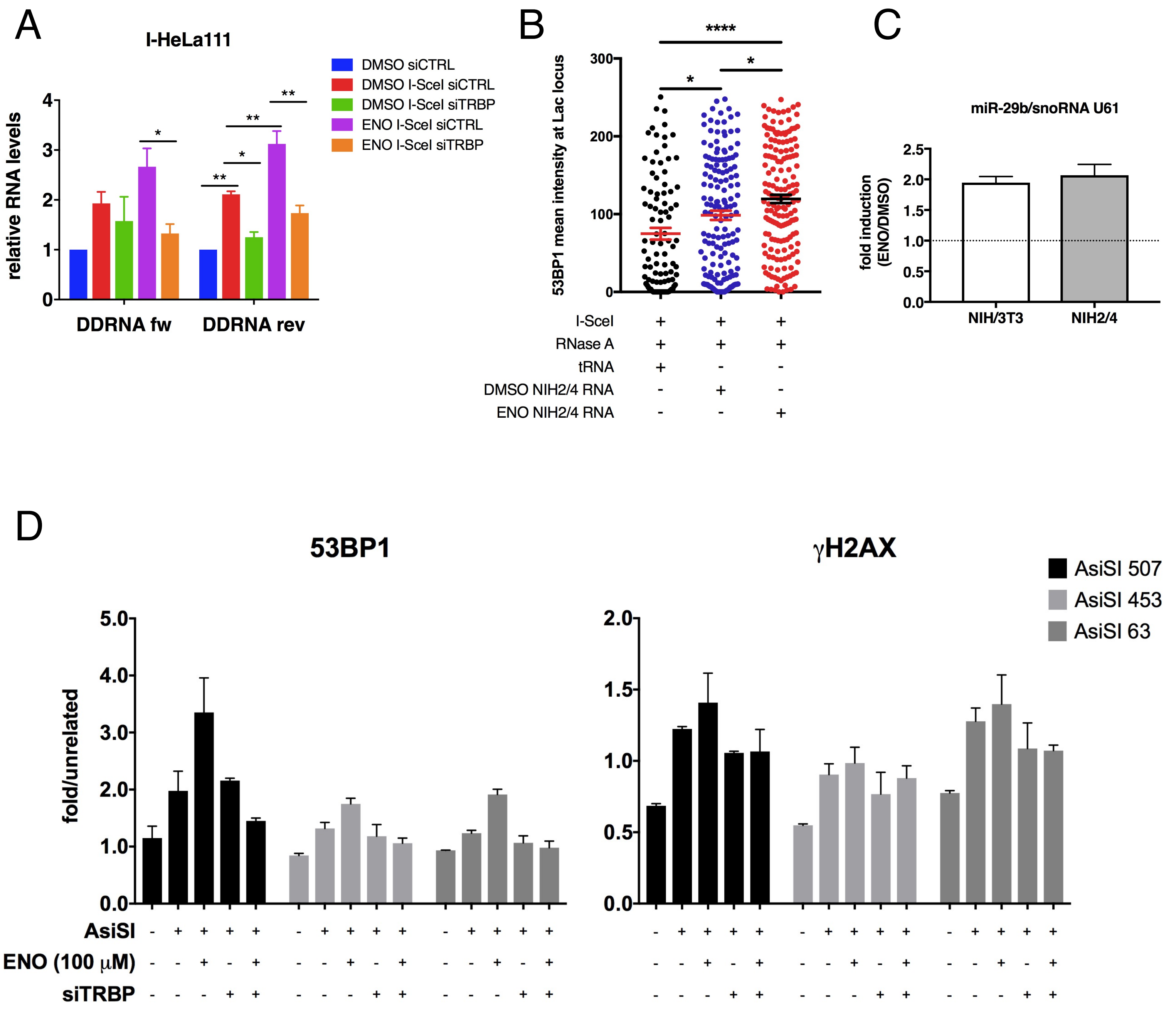

A
I-HeLa111
relative RNA levels
DDRNA fw
DDRNA rev
DMSO siCTRL
DMSO I-SceI siCTRL
DMSO I-SceI siTRBP
ENO I-SceI siCTRL
ENO I-SceI siTRBP
B
53BP1 mean intensity at Lac locus
I-SceI
RNase A
tRNA
DMSO NIH2/4 RNA
ENO NIH2/4 RNA
C
miR-29b/snoRNA U61
fold induction (ENO/DMSO)
NIH/3T3
NIH2/4
D
53BP1
γH2AX
fold/unrelated
AsiSI 507
AsiSI 453
AsiSI 63
AsiSI
ENO (100 μM)
siTRBP

Figure S4. (**A**) I-HeLa111 cells were transfected with siRNAs against TRBP (siTRBP) or with a non-targeting siRNA as a control (siCTRL). 48 hours later, cells were incubated with 1 μg ml^{-1} doxycycline to induce I-SceI expression and simultaneously treated with 150 μM enoxacin for other 24 hours. Small RNAs were gel-extracted and analysed by qRT-PCR as in Figure 4A. Values in the histograms are the averages ± s.e.m. of three independent experiments. (**B**) The dot plot shows the intensity of 53BP1 foci at Lac locus from the samples represented at lanes 2-4 of Fig. 4C. (**C**) qRT-PCR analysis of miR-29b expression in enoxacin-treated NIH/3T3 and NIH2/4 cells. Histogram shows miR-29b levels relative to snoRNA U61 used as a loading control. Values are normalised to DMSO-treated samples and shown as the averages ± s.e.m. of three independent experiments. (**D**) AsiSI-ER U2OS cells were transfected with siRNAs against TRBP (siTRBP+) or with a non-targeting siRNA as a control (siTRBP-). 24 h after transfection, cells were incubated or not with 100 μM enoxacin (ENO+) for other 24 h prior to AsiSI cleavage induction, as described in (Figure 4D). Values are normalised as in (Figure 4D) and represent the means ± s.e.m. of at least two independent experiments.

Figure S5

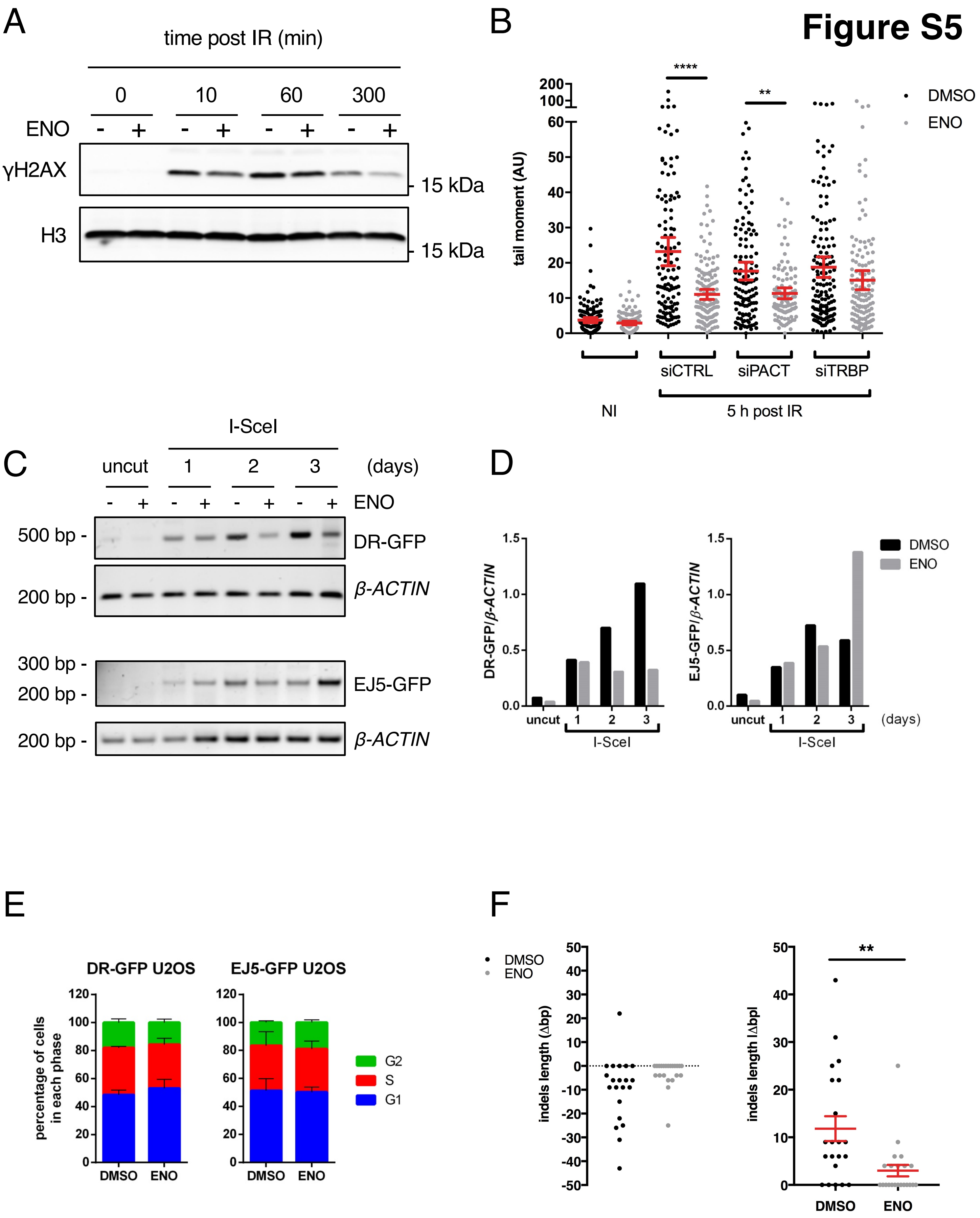

A
time post IR (min)
0
10
60
300
ENO
- + - + - + - +
γH2AX
H3
15 kDa
15 kDa
B
tail moment (AU)
****
**
DMSO
ENO
siCTRL
siPACT
siTRBP
NI
5 h post IR
C
I-SceI
uncut
1
2
3
(days)
ENO
500 bp -
200 bp -
300 bp -
200 bp -
200 bp -
DR-GFP
β-ACTIN
EJ5-GFP
β-ACTIN
D
DR-GFP/β-ACTIN
EJ5-GFP/β-ACTIN
DMSO
ENO
uncut
1
2
3
I-SceI
(days)
E
DR-GFP U2OS
EJ5-GFP U2OS
percentage of cells in each phase
G2
S
G1
DMSO
ENO
F
DMSO
ENO
indels length (Δbp)
indels length |Δbp|
**
DMSO
ENO

Figure S5. (**A**) Full-length version of the blot shown in Figure 5B. (**B**) 48 hours prior to IR, HeLa cells were transfected with siRNAs against PACT (siPACT), TRBP (siTRBP) or with non-targeting siRNA (siCTRL) and simultaneously treated with 50 μM enoxacin. 5 hours post IR, cells were analysed by neutral comet assay along with not irradiated cells (NI). The dot-plot shows comet tail moment. Red bars represent the averages ± 95% CI of three independent experiments; at least 100 cells per sample were scored. (**C**) Representative pictures of PCR products fractionated on agarose gels from the assays described in Figure 5D. PCR with primers spanning I-SceI cut sites were performed on genomic DNA collected at 1, 2 and 3 days after I-SceI expression. Uncut cells were also examined. *β-ACTIN* gene DNA was used as a normaliser. (**D**) Densitometric analysis of PCR products shown in (C). (**E**) Cell cycle profile was determined by flow-cytometry on cells treated with enoxacin (ENO) or DMSO for 3 days. Values are the means ± s.e.m. of at least two independent experiments. (**F**) Dot plots showing the length of indels occurring at the I-SceI junction of re-joined DNA fragments from the experiment described in Figure 5E. Values are shown as both real and absolute numbers (left and right panel, respectively). Red-bars represent the averages ± s.e.m. of 3 independent biological replicates. At least 20 clones for sample were analysed.

Figure S6

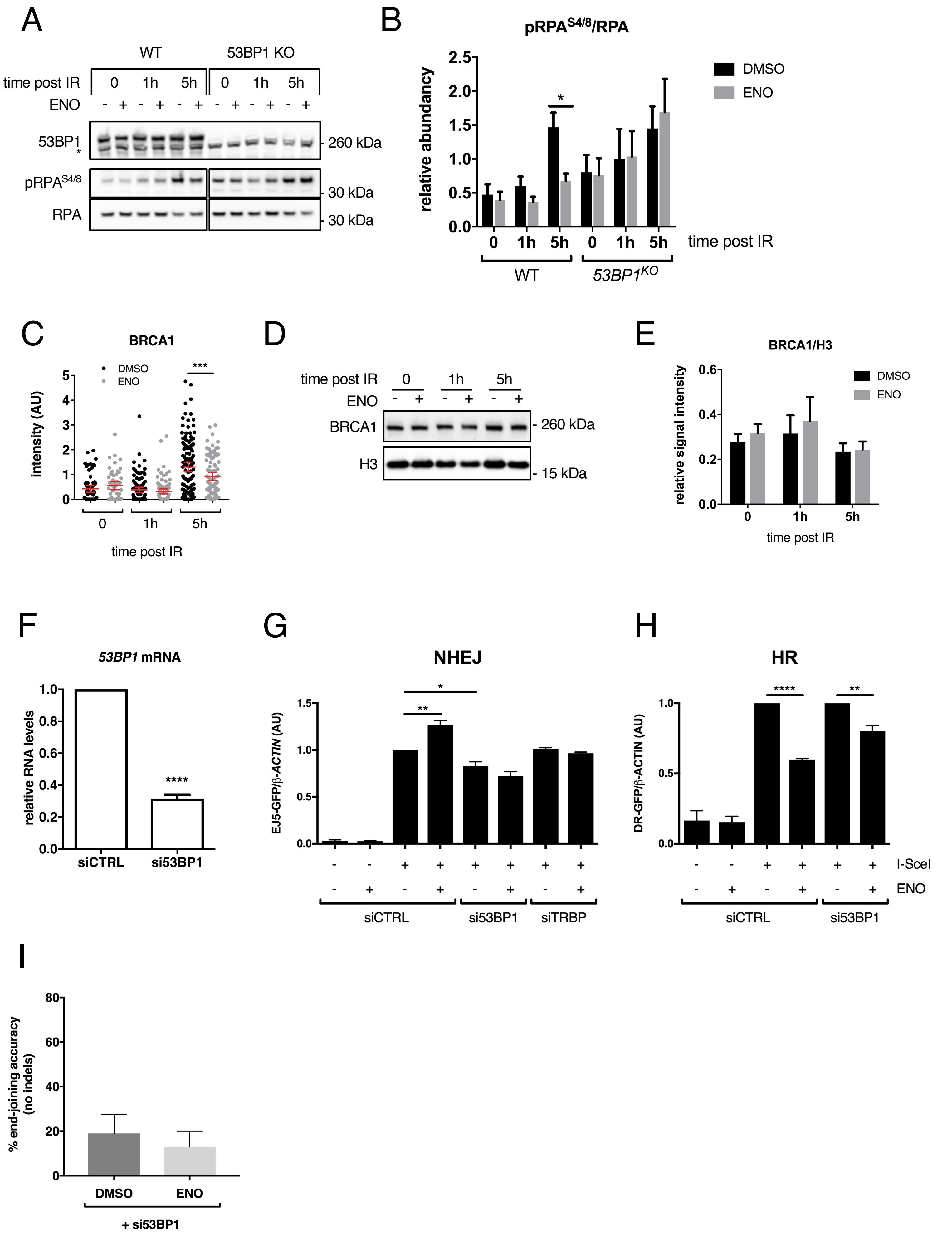

A
WT
53BP1 KO
time post IR
0
1h
5h
ENO
53BP1
260 kDa
pRPA S4/8
30 kDa
RPA
30 kDa
B
pRPA S4/8/RPA
relative abundancy
DMSO
ENO
time post IR
WT
53BP1 KO
C
BRCA1
intensity (AU)
DMSO
ENO
***
time post IR
D
time post IR
ENO
BRCA1
260 kDa
H3
15 kDa
E
BRCA1/H3
relative signal intensity
DMSO
ENO
time post IR
F
53BP1 mRNA
relative RNA levels
****
siCTRL
si53BP1
G
NHEJ
EJ5-GFP/β-ACTIN (AU)
siCTRL
si53BP1
siTRBP
H
HR
DR-GFP/β-ACTIN (AU)
I-SceI
ENO
siCTRL
si53BP1
I
% end-joining accuracy (no indels)
DMSO
ENO
+ si53BP1

Figure S6

J

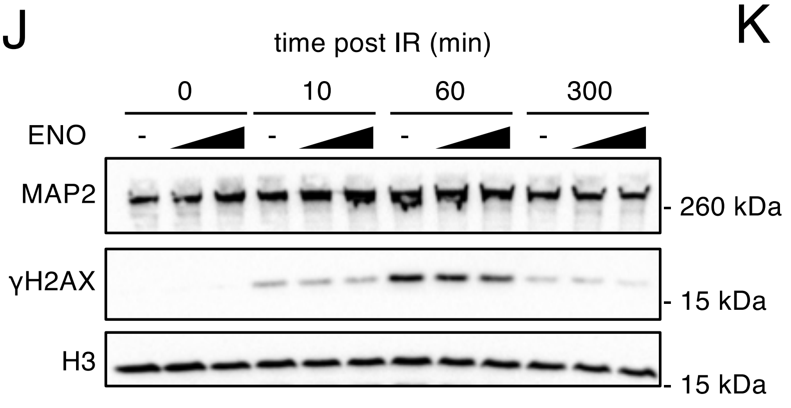

K

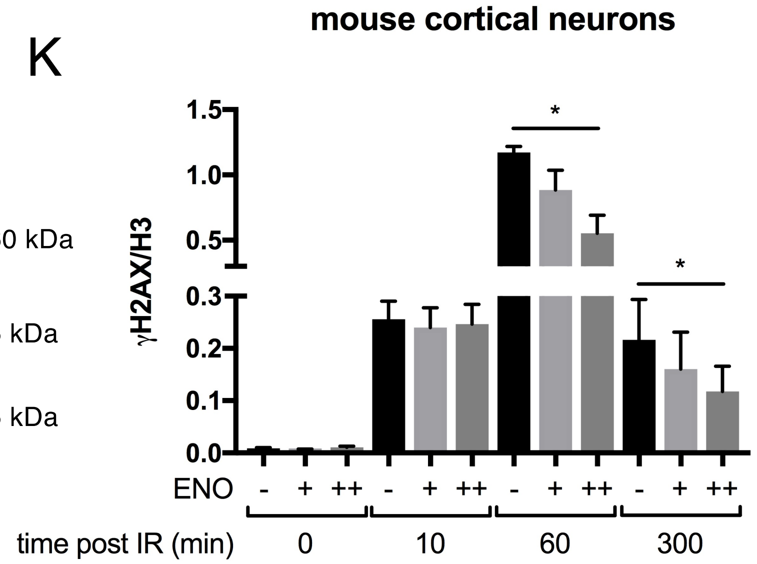

L

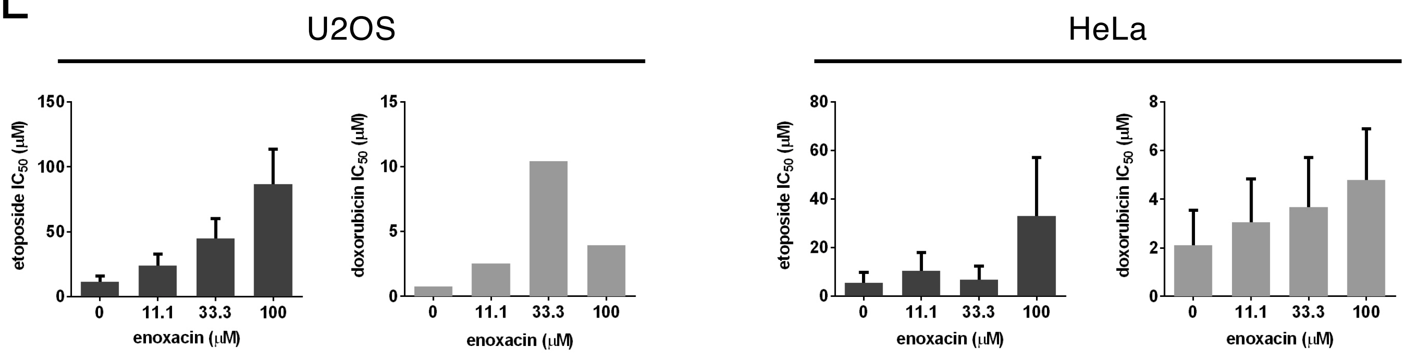

Figure S6. (**A**) Whole protein lysates from U2OS cells treated as in Figure 6B were probed for the indicated proteins by immunoblotting. The asterisk marks unspecific bands. (**B**) Densitometric analysis of $pRPA^{S4/8}$ relative to RPA. Values are the averages ± s.e.m. of at least three independent experiments (Student's t-test). (**C**) U2OS cells were treated with 50 μM enoxacin for 48 h prior to IR, fixed 1 h and 5 h post IR and stained for BRCA1. The dot plot shows the intensity of BRCA1 foci per nucleus of untreated (black dots) and enoxacin-treated (grey dots) cells. Red bars are the averages ± 95% CI of three independent experiments; at least 100 cells per sample were scored. (**D**) Representative immuno-blot of total cell lysates from (C) probed for BRCA1 and H3 as a loading control. (**E**) Densitometric analysis of BRCA1 relative to H3 represented in (D). Values are the averages ± s.e.m. of three independent experiments. (**F**) DR-GFP or EJ5-GFP U2OS cells were transfected with the indicated siRNAs. Total RNA was purified and analysed 48 hours after transfection. Histograms show the averages ± s.e.m. from three independent experiments (Student's t-test). (**G, H**) EJ5-GFP (G) or DR-GFP (H) U2OS cells were transfected with siRNAs against 53BP1 (+si53BP1) for 48 hours prior to enoxacin administration (+ENO) and I-SceI expression (+I-SceI). Histograms show results of qPCR performed with primers spanning I-SceI cut sites on gDNA collected 72 hours after I-SceI expression. *β-ACTIN* gene DNA was used as a normaliser. Values are relative to cut DMSO-treated cells (+I-SceI, -ENO) and represented as the means ± s.e.m. of three independent experiments (Student's t-test). (**I**) gDNA extracted from 53BP1-depleted EJ5-GFP U2OS cells was analysed by Sanger sequencing for the presence of indels at the I-SceI site as in Figure 5E. The histogram shows the percentage of re-joining events containing no indels from DMSO or enoxacin (ENO) treated cells. Values are the averages ± s.e.m. of three independent experiments. At least 20 clones for sample were analysed. (**J**) Total protein lysates from mouse cortical neurons, incubated with 50 or 150 μM enoxacin for 48 hours before IR, were analysed by immunoblotting at different time-points post IR. Microtubule-associated protein 2 (MAP2) was studied as a neuronal-specific marker. (**K**) Densitometric analysis of γH2AX signal relative to H3 in mouse cortical neurons treated as in (J); values are shown as the averages ± s.e.m. of three independent experiments. (**L**) Biological replicate of the experiment shown in Figure 6C.

Table S1

Oligonucleotides	Company	Catalogue number
siRNAs against human 53BP1	Dharmacon	L-003548
siRNAs against human TARBP2	Dharmacon	LQ-017430
siRNAs against human PACT	Dharmacon	LQ-006426
siRNAs against human TNRC6A	Dharmacon	D-014107-01/02
siRNAs against human TNRC6B	Dharmacon	D-024575-02/17
siRNAs against human TNRC6C	Dharmacon	D-019399-01/02
siRNA against GFP	Dharmacon	P-002048-01
non-targeting siRNA pool	Dharmacon	D-001810-10
primers for human TARBP2 mRNA (F: GGCCCTCAAACACCTCAA, R: GTCCTCAGGCAGTGAAGAGTC)	This work	N/A
primers for human PACT mRNA (F: CTCCAGAGAACCACATTTCTTTAAC, R: GGAGGCTTCTTTTCAGTAAGTTGAT)	This work	N/A
primers for human TNRC6A mRNA (F: AACAAAGAGGCAAGCAGTGG, R: CCCCATGCTGAAGTACCATTA)	This work	N/A
primers for human TNRC6B mRNA (F: GGTGGCTCAGTTCGTCCTAGT, R: TCTCAAGGTTGACCCATCAAT)	This work	N/A
primers for human TNRC6C mRNA (F: CCCAGTAAGCTCCAACCAGA, R: CAGTTGGCTTCGCTTCTGTA)	This work	N/A
primers for human B2M mRNA (F: TTCTGGCCTGGAGGCTATC, R: TCAGGAAATTTGACTTTCCATTC)	This work	N/A
synthetic cel-miR-67* (CGCUCAUUCUGCCGGUUGUUAUG)	This work	N/A
primer for cel-miR-67* (CGCTCATTCTGCCGGTTGTTATG)	This work	N/A
primer for miR-29b (TAGCACCATTTGAAATCAGTGTT)	This work	N/A
primer for DDRNA fw (TCCACATGTGGCCACAAATTG)	This work	N/A
primer for DDRNA rev (CAATTTGTGGCCACATGTGGA)	This work	N/A
primer for Telo G	Rossiello, 2017 Nat Comm	N/A
primer for Telo C	Rossiello, 2017 Nat Comm	N/A
primer for U61 snoRNA	Qiagen	MS00033705
primers for DRGFP genomic locus (F: GAGGGCGAGGGCGATGCC, R: TGCACGCTGCCGTCCTCG)	This work	N/A
primers for EJ5GFP genomic locus (F: CTTTTTCCTACAGCTCCTGGGCA, R: GGTGGTGCAGATGAACTTCAGGG)	This work	N/A
primers for human beta-Actin gene (F: GATCATTGCTCCTCCTGAGC, R: AAAGCCATGCCAATCTCATC)	This work	N/A
ChIP primers for AsiSI 507 (F: GATTGGCTATGGGTGTGGAC, R: CATCCTTGCAAACCAGTCCT)	This work	N/A
ChIP primers for AsiSI 453 (F: GGCGTACTGGTGGAAAGC, R: TCAGAGTCCGAATACTTAACTACGG)	This work	N/A
ChIP primers for AsiSI 63 (F: AGGCTGCTTGGGATTTAGGC, R: TCCCAGCCCACCTTCATTTG)	This work	N/A
ChIP primers for the unrelated region (F: CAACGCCGAGATCTCCAA, R: GGATCTTGTCGCTGTCTTTGA)	This work	N/A

Table S1. The table shows RNA and DNA oligonucleotides used in this work.

Table S2

Antibody	Company	Catalogue number	Dilution
Mouse monoclonal anti-ATM	Sigma-Aldrich	A1106	1:1000
Mouse monoclonal anti-BRCA1	Santa Cruz Biotechnology	sc-6954	1:500
Mouse monoclonal anti-BrdU	GE Healthcare	RPN20AB	1:800
Mouse monoclonal anti-CHK2	Millipore	05-649	1:1000
Mouse monoclonal anti-histone H3	Abcam	ab10799	1:3000
Mouse monoclonal anti-MDC1	Sigma-Aldrich	M2444	1:500
Mouse monoclonal anti-P53	Abcam	ab1101	1:500
Mouse monoclonal anti-PACT	Santa Cruz Biotechnology	sc-377103	1:500
Mouse monoclonal anti-phospho ATM (Ser1981)	Rockland	200-301-400	1:1000
Mouse monoclonal anti-RPA	Calbiochem	NA18-100UG	1:1000
Mouse monoclonal anti-TRBP	Sigma-Aldrich	SAB1406507	1:500
Mouse monoclonal anti-Tubulin	Sigma-Aldrich	T5168	1:5000
Mouse monoclonal anti-Vinculin	Sigma-Aldrich	V9131	1:5000
Mouse monoclonal anti-γH2AX (Ser139)	Millipore	05-636	1:1000
Rabbit monoclonal anti-γH2AX (Ser139)	Abcam	ab81299	ChIP
Rabbit polyclonal anti-53BP1	Novus-Biologicals	NB100-304	1:1000
Rabbit polyclonal anti-53BP1	Novus-Biologicals	NB100-305	ChIP
Rabbit polyclonal anti-Microtubule-Associated Protein 2 (MAP2)	Millipore	AB5622	1:3000
Rabbit polyclonal anti-phospho RPA32 (S4/S8)	Bethyl	A300-245A	1:500
Rabbit polyclonal anti-phospho-(Ser/Thr) ATM/ATR substrate	Cell Signaling	#2851	1:200
Rabbit polyclonal anti-phospho-CHK2 (Thr68)	Cell Signaling	#2661	1:1000
Rabbit polyclonal anti-phospho-P53 (ser15)	Cell Signaling	#9284	1:500

Table S2. The table lists the primary antibodies used for immunofluorescence, immunoblot and ChIP analyses.
